# Supplementary material for: Mycobacterium bovis Infection Frequently Requires Surgical Intervention in Individuals with HIV
Source: Infect Dis Rep. 2025 Jul 11;17(4):82. doi: 10.3390/idr17040082 (PMC12286270; doi:10.3390/idr17040082)
Supplement: Supplementary file 1 [file idr-17-00082-s001.zip › idr-3629666-supplementary.pdf]

Sociodemographic characteristics of patients with M tuberculosis or M. bovis infection in HIV-infected subjects seen at HCGFAA.

Andrea Torres Rojas

2025-05-30

```
library(readxl)
Data <- read_excel("C:/Users/andre/OneDrive/Escritorio/Data.xlsx")
head(Data)
```

```
## # A tibble: 6 x 15
##   Groups    ID   Age   Sex Residence Alcoholism Smoking `Contact with cattle`
##    <dbl> <dbl> <dbl> <dbl>     <dbl>      <dbl>   <dbl>                 <dbl>
## 1      1     1    48     1         1          1       1                     2
## 2      1     2    41     1         1          2       2                     2
## 3      1     3    56     1         1          2       2                     2
## 4      1     4    38     1         1          1       1                     2
## 5      1     5    40     1         1          2       2                     2
## 6      1     6    31     1         1          1       1                     2
## # ... with 7 more variables: Contact with TB <dbl>, Trace work <dbl>,
## #   Regular consumption of unpasteurized dairy products <dbl>, T2D <dbl>,
## #   Charlson <dbl>, Lymphocytes CD4 Count <dbl>, Viral Loads <dbl>
```

```
Data$Groups<-as.factor(Data$Groups)
Data$Groups<-factor(Data$Groups,levels = c(1,2), labels = c("M.Bovis", "M.TB"))
Data$Sex<-as.factor(Data$Sex)
Data$Sex<-factor(Data$Sex, levels= c(1,2), labels =c("masculine","femenine"))
Data$Residence<-as.factor(Data$Residence)
Data$Residence<-factor(Data$Residence, levels = c(1,2), labels = c("urban", "rural"))
Data$Alcoholism<-as.factor(Data$Alcoholism)
Data$Alcoholism<-factor(Data$Alcoholism, levels= c(1,2), labels =c("yes","no"))
Data$Smoking<-as.factor(Data$Smoking)
Data$Smoking<-factor(Data$Smoking, levels= c(1,2), labels =c("yes","no"))
Data$`Contact with cattle`<-as.factor(Data$`Contact with cattle`)
Data$`Contact with cattle`<-factor(Data$`Contact with cattle`, levels= c(1,2), labels =c("yes","no"))
Data$`Contact with TB`<-as.factor(Data$`Contact with TB`)
Data$`Contact with TB`<-factor(Data$`Contact with TB`, levels= c(1,2), labels =c("yes","no"))
Data$`Trace work`<-as.factor(Data$`Trace work`)
Data$`Trace work`<-factor(Data$`Trace work`, levels= c(1,2), labels =c("yes","no"))
Data$`Regular consumption of unpasteurized dairy products`<-as.factor(Data$`Regular consumption of unpas
Data$`Regular consumption of unpasteurized dairy products`<-factor(Data$`Regular consumption of unpasteu
Data$T2D<-as.factor(Data$T2D)
Data$T2D<-factor(Data$T2D, levels= c(1,2), labels =c("yes","no"))
```

```r
str(Data)
```

```
## tibble [26 x 15] (S3: tbl_df/tbl/data.frame)
##  $ Groups                                             : Factor w/ 2 levels "M.Bovis","M.TB": 1 1 1 1
##  $ ID                                                 : num [1:26] 1 2 3 4 5 6 7 8 9 10 ...
##  $ Age                                                : num [1:26] 48 41 56 38 40 31 46 43 30 25 ...
##  $ Sex                                                : Factor w/ 2 levels "masculine","femenine": 1
##  $ Residence                                          : Factor w/ 2 levels "urban","rural": 1 1 1 1
##  $ Alcoholism                                         : Factor w/ 2 levels "yes","no": 1 2 2 1 2 1 2
##  $ Smoking                                            : Factor w/ 2 levels "yes","no": 1 2 2 1 2 1 2
##  $ Contact with cattle                                : Factor w/ 2 levels "yes","no": 2 2 2 2 2 2 2
##  $ Contact with TB                                    : Factor w/ 2 levels "yes","no": 1 2 2 2 2 2 2
##  $ Trace work                                         : Factor w/ 2 levels "yes","no": 2 2 2 2 2 2 2
##  $ Regular consumption of unpasteurized dairy products: Factor w/ 2 levels "yes","no": 1 1 1 1 2 2 1
##  $ T2D                                                : Factor w/ 2 levels "yes","no": 2 2 2 2 1 2 2
##  $ Charlson                                           : num [1:26] 6 6 8 8 7 6 6 6 6 6 ...
##  $ Lymphocytes CD4 Count                              : num [1:26] 177 108 97 150 44 238 170 30 17 1
##  $ Viral Loads                                        : num [1:26] 301 588673 19 152 115000 ...
```

#Analysis Table 1 ##Analysis Categorical Variables

```r
Table.Frequencies <- function(x){
  y <- table(x)
  data_freq <-
    data.frame(Niveles= names(y),
               Freq =as.numeric(y),
               Freq_Rela = as.numeric(prop.table(y)),
               Freq_Acum = as.numeric(cumsum(y)),
               Freq_Rela_Acum = as.numeric (cumsum(prop.table(y))))

  data_freq}
```

```r
table(Data$Sex, Data$Groups)
```

```
##
##              M.Bovis M.TB
##   masculine      11   14
##   femenine        1    0
```

```r
x<-table(Data$Sex, Data$Groups)
fisher.test(x)
```

```
##
##  Fisher's Exact Test for Count Data
##
## data:  x
## p-value = 0.4615
## alternative hypothesis: true odds ratio is not equal to 1
## 95 percent confidence interval:
##   0.00000 33.42891
## sample estimates:
## odds ratio
##          0
```

```r
gr <- split(Data$Sex, Data$Groups)
results <- lapply(gr, Table.Frequencies)
data.frame(results)
```

```
##   M.Bovis.Niveles M.Bovis.Freq M.Bovis.Freq_Rela M.Bovis.Freq_Acum
## 1       masculine           11        0.91666667                11
## 2        femenine            1        0.08333333                12
##   M.Bovis.Freq_Rela_Acum M.TB.Niveles M.TB.Freq M.TB.Freq_Rela M.TB.Freq_Acum
## 1              0.9166667    masculine        14              1             14
## 2              1.0000000     femenine         0              0             14
##   M.TB.Freq_Rela_Acum
## 1                   1
## 2                   1
```

```r
table(Data$Residence, Data$Groups)
```

```
##
##         M.Bovis M.TB
##   urban      12   14
##   rural       0    0
```

```r
x<-table(Data$Residence, Data$Groups)
fisher.test(x)
```

```
##
##  Fisher's Exact Test for Count Data
##
## data:  x
## p-value = 1
## alternative hypothesis: true odds ratio is not equal to 1
## 95 percent confidence interval:
##    0 Inf
## sample estimates:
## odds ratio
##          0
```

```r
gr <- split(Data$Residence, Data$Groups)
results <- lapply(gr, Table.Frequencies)
data.frame(results)
```

```
##   M.Bovis.Niveles M.Bovis.Freq M.Bovis.Freq_Rela M.Bovis.Freq_Acum
## 1           urban           12                 1                12
## 2           rural            0                 0                12
##   M.Bovis.Freq_Rela_Acum M.TB.Niveles M.TB.Freq M.TB.Freq_Rela M.TB.Freq_Acum
## 1                      1        urban        14              1             14
## 2                      1        rural         0              0             14
##   M.TB.Freq_Rela_Acum
## 1                   1
## 2                   1
```

```r
table(Data$Alcoholism, Data$Groups)
```

```
##
##       M.Bovis M.TB
##   yes       6    7
##   no        6    7
```

```r
x<-table(Data$Alcoholism, Data$Groups)
fisher.test(x)
```

```
##
##  Fisher's Exact Test for Count Data
##
## data:  x
## p-value = 1
## alternative hypothesis: true odds ratio is not equal to 1
## 95 percent confidence interval:
##  0.1657209 6.0342427
## sample estimates:
## odds ratio
##          1
```

```r
gr <- split(Data$Alcoholism, Data$Groups)
results <- lapply(gr, Table.Frequencies)
data.frame(results)
```

```
##   M.Bovis.Niveles M.Bovis.Freq M.Bovis.Freq_Rela M.Bovis.Freq_Acum
## 1             yes            6               0.5                 6
## 2              no            6               0.5                12
##   M.Bovis.Freq_Rela_Acum M.TB.Niveles M.TB.Freq M.TB.Freq_Rela M.TB.Freq_Acum
## 1                    0.5          yes         7            0.5              7
## 2                    1.0           no         7            0.5             14
##   M.TB.Freq_Rela_Acum
## 1                 0.5
## 2                 1.0
```

```r
table(Data$Smoking, Data$Groups)
```

```
##
##       M.Bovis M.TB
##   yes       4   10
##   no        8    4
```

```r
x<-table(Data$Smoking, Data$Groups)
fisher.test(x)
```

```
##
##  Fisher's Exact Test for Count Data
##
## data:  x
```

```
## p-value = 0.1131
## alternative hypothesis: true odds ratio is not equal to 1
## 95 percent confidence interval:
##  0.0272772 1.3606514
## sample estimates:
## odds ratio
##  0.2142874
```

```
gr <- split(Data$Smoking, Data$Groups)
results <- lapply(gr, Table.Frequencies)
data.frame(results)
```

```
##   M.Bovis.Niveles M.Bovis.Freq M.Bovis.Freq_Rela M.Bovis.Freq_Acum
## 1             yes            4         0.3333333                 4
## 2              no            8         0.6666667                12
##   M.Bovis.Freq_Rela_Acum M.TB.Niveles M.TB.Freq M.TB.Freq_Rela M.TB.Freq_Acum
## 1              0.3333333          yes        10      0.7142857             10
## 2              1.0000000           no         4      0.2857143             14
##   M.TB.Freq_Rela_Acum
## 1           0.7142857
## 2           1.0000000
```

```
table(Data$`Contact with cattle`, Data$Groups)
```

```
##
##       M.Bovis M.TB
##   yes       0    0
##   no       12   14
```

```
x<-table(Data$`Contact with cattle`, Data$Groups)
fisher.test(x)
```

```
##
##  Fisher's Exact Test for Count Data
##
## data:  x
## p-value = 1
## alternative hypothesis: true odds ratio is not equal to 1
## 95 percent confidence interval:
##    0 Inf
## sample estimates:
## odds ratio
##          0
```

```
gr <- split(Data$`Contact with cattle`, Data$Groups)
results <- lapply(gr, Table.Frequencies)
data.frame(results)
```

```
##   M.Bovis.Niveles M.Bovis.Freq M.Bovis.Freq_Rela M.Bovis.Freq_Acum
## 1             yes            0                 0                 0
## 2              no           12                 1                12
```

```
##   M.Bovis.Freq_Rela_Acum M.TB.Niveles M.TB.Freq M.TB.Freq_Rela M.TB.Freq_Acum
## 1                      0          yes         0              0              0
## 2                      1           no        14              1             14
##   M.TB.Freq_Rela_Acum
## 1                   0
## 2                   1
```

```
table(Data$`Contact with TB`,Data$Groups)
```

```
##
##       M.Bovis M.TB
##   yes       1    1
##   no       11    9
```

```
x<-table(Data$`Contact with TB`, Data$Groups)
fisher.test(x)
```

```
##
##  Fisher's Exact Test for Count Data
##
## data:  x
## p-value = 1
## alternative hypothesis: true odds ratio is not equal to 1
## 95 percent confidence interval:
##   0.009573596 71.239880135
## sample estimates:
## odds ratio
##  0.8257258
```

```
gr <- split(Data$`Contact with TB`, Data$Groups)
results <- lapply(gr, Table.Frequencies)
data.frame(results)
```

```
##   M.Bovis.Niveles M.Bovis.Freq M.Bovis.Freq_Rela M.Bovis.Freq_Acum
## 1             yes            1        0.08333333                 1
## 2              no           11        0.91666667                12
##   M.Bovis.Freq_Rela_Acum M.TB.Niveles M.TB.Freq M.TB.Freq_Rela M.TB.Freq_Acum
## 1             0.08333333          yes         1            0.1              1
## 2             1.00000000           no         9            0.9             10
##   M.TB.Freq_Rela_Acum
## 1                 0.1
## 2                 1.0
```

```
table(Data$`Trace work`,Data$Groups)
```

```
##
##       M.Bovis M.TB
##   yes       0    0
##   no       12   14
```

```r
x<-table(Data$`Trace work`, Data$Groups)
fisher.test(x)
```

```
##
##  Fisher's Exact Test for Count Data
##
## data:  x
## p-value = 1
## alternative hypothesis: true odds ratio is not equal to 1
## 95 percent confidence interval:
##    0 Inf
## sample estimates:
## odds ratio
##          0
```

```r
gr <- split(Data$`Trace work`, Data$Groups)
results <- lapply(gr, Table.Frequencies)
data.frame(results)
```

```
##   M.Bovis.Niveles M.Bovis.Freq M.Bovis.Freq_Rela M.Bovis.Freq_Acum
## 1             yes            0                 0                 0
## 2              no           12                 1                12
##   M.Bovis.Freq_Rela_Acum M.TB.Niveles M.TB.Freq M.TB.Freq_Rela M.TB.Freq_Acum
## 1                      0          yes         0              0              0
## 2                      1           no        14              1             14
##   M.TB.Freq_Rela_Acum
## 1                   0
## 2                   1
```

```r
table(Data$`Regular consumption of unpasteurized dairy products`,Data$Groups)
```

```
##
##       M.Bovis M.TB
##   yes       9    0
##   no        3   14
```

```r
x<-table(Data$`Regular consumption of unpasteurized dairy products`, Data$Groups)
fisher.test(x)
```

```
##
##  Fisher's Exact Test for Count Data
##
## data:  x
## p-value = 7.041e-05
## alternative hypothesis: true odds ratio is not equal to 1
## 95 percent confidence interval:
##  5.002175      Inf
## sample estimates:
## odds ratio
##        Inf
```

```
gr <- split(Data$`Regular consumption of unpasteurized dairy products`, Data$Groups)
results <- lapply(gr, Table.Frequencies)
data.frame(results)
```

```
##   M.Bovis.Niveles M.Bovis.Freq M.Bovis.Freq_Rela M.Bovis.Freq_Acum
## 1             yes            9              0.75                 9
## 2              no            3              0.25                12
##   M.Bovis.Freq_Rela_Acum M.TB.Niveles M.TB.Freq M.TB.Freq_Rela M.TB.Freq_Acum
## 1                   0.75          yes         0              0              0
## 2                   1.00           no        14              1             14
##   M.TB.Freq_Rela_Acum
## 1                   0
## 2                   1
```

```
table(Data$T2D,Data$Groups)
```

```
##
##       M.Bovis M.TB
##   yes       1    0
##   no       11   14
```

```
x<-table(Data$T2D, Data$Groups)
fisher.test(x)
```

```
##
##  Fisher's Exact Test for Count Data
##
## data:  x
## p-value = 0.4615
## alternative hypothesis: true odds ratio is not equal to 1
## 95 percent confidence interval:
##  0.02991422        Inf
## sample estimates:
## odds ratio
##        Inf
```

```
gr <- split(Data$T2D, Data$Groups)
results <- lapply(gr, Table.Frequencies)
data.frame(results)
```

```
##   M.Bovis.Niveles M.Bovis.Freq M.Bovis.Freq_Rela M.Bovis.Freq_Acum
## 1             yes            1        0.08333333                 1
## 2              no           11        0.91666667                12
##   M.Bovis.Freq_Rela_Acum M.TB.Niveles M.TB.Freq M.TB.Freq_Rela M.TB.Freq_Acum
## 1             0.08333333          yes         0              0              0
## 2             1.00000000           no        14              1             14
##   M.TB.Freq_Rela_Acum
## 1                   0
## 2                   1
```

##Analysis Numerical Variables

```r
normality<-function(x){
  car::qqPlot(x)
  boxplot(x)
  a<-shapiro.test(x)
  b<-FSA::ksTest(x,"pnorm", mean(x), sd(x))
  print(a)
  print(b)
  }
```

```r
normality(Data$Age)
```

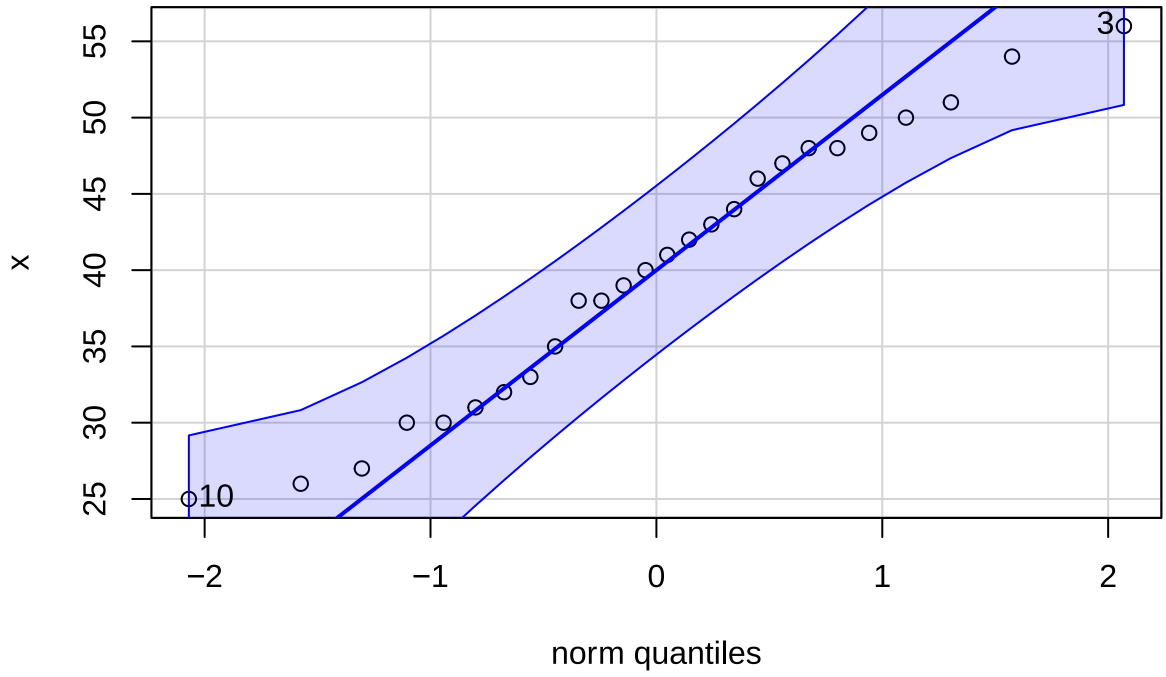


```
## Registered S3 methods overwritten by 'FSA':
##   method       from
##   confint.boot car
##   hist.boot    car
```

```
## Warning in stats::ks.test(x, y, ..., alternative = alternative, exact = exact):
## ties should not be present for the Kolmogorov-Smirnov test
```

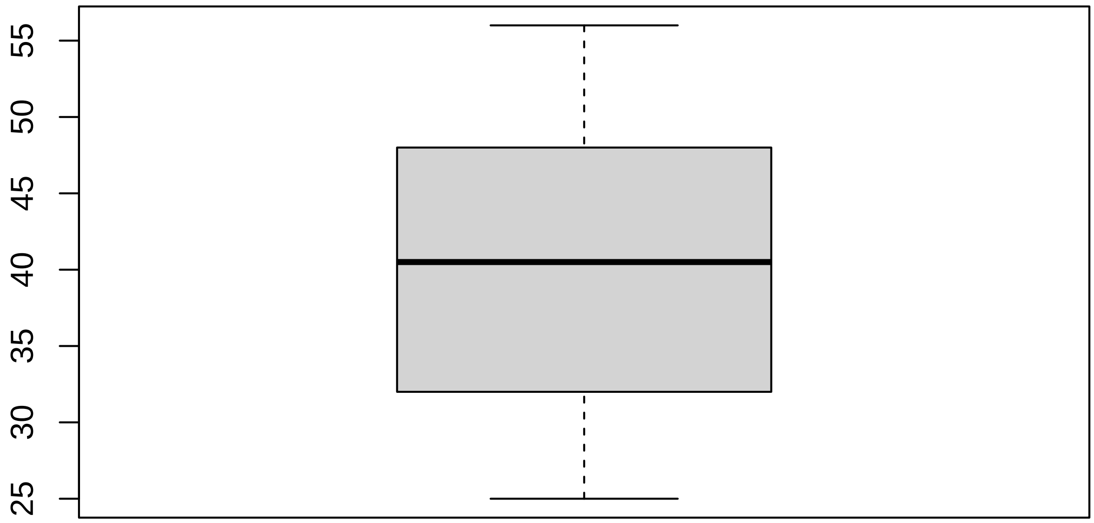


```
##
##  Shapiro-Wilk normality test
##
## data:  x
## W = 0.9649, p-value = 0.4969
##
##
##  One-sample Kolmogorov-Smirnov test
##
## data:  x
## D = 0.093694, p-value = 0.9764
## alternative hypothesis: two-sided
```

```
normality(Data$Charlson)
```

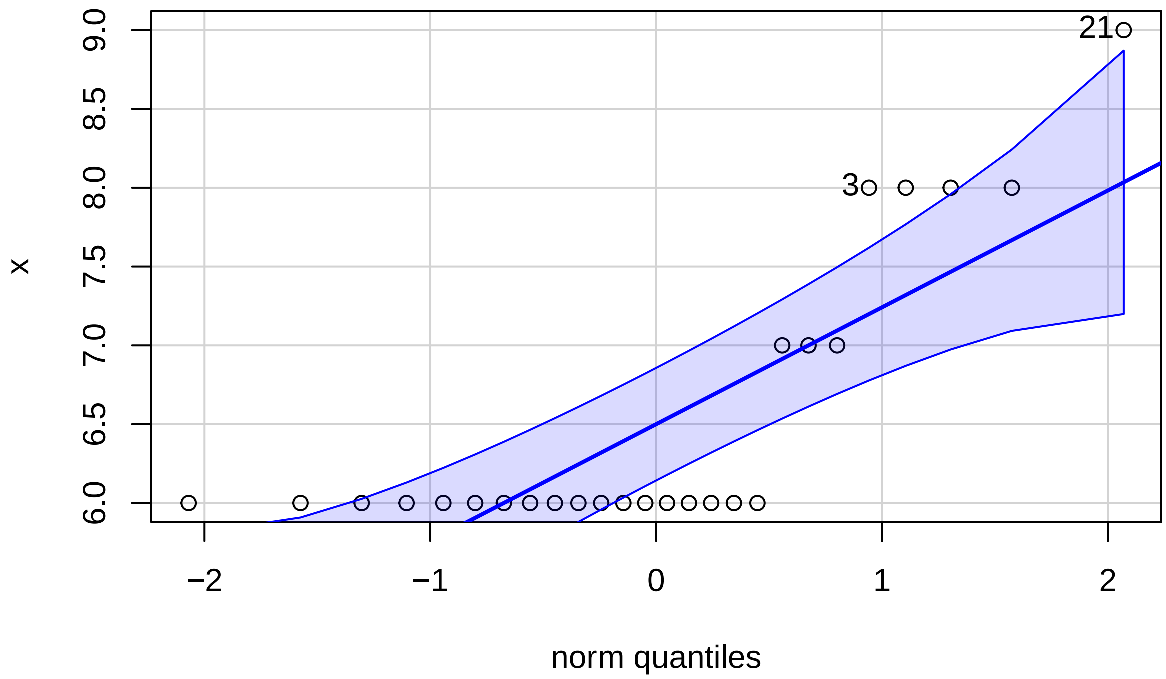


```
## Warning in stats::ks.test(x, y, ..., alternative = alternative, exact = exact):
## ties should not be present for the Kolmogorov-Smirnov test
```

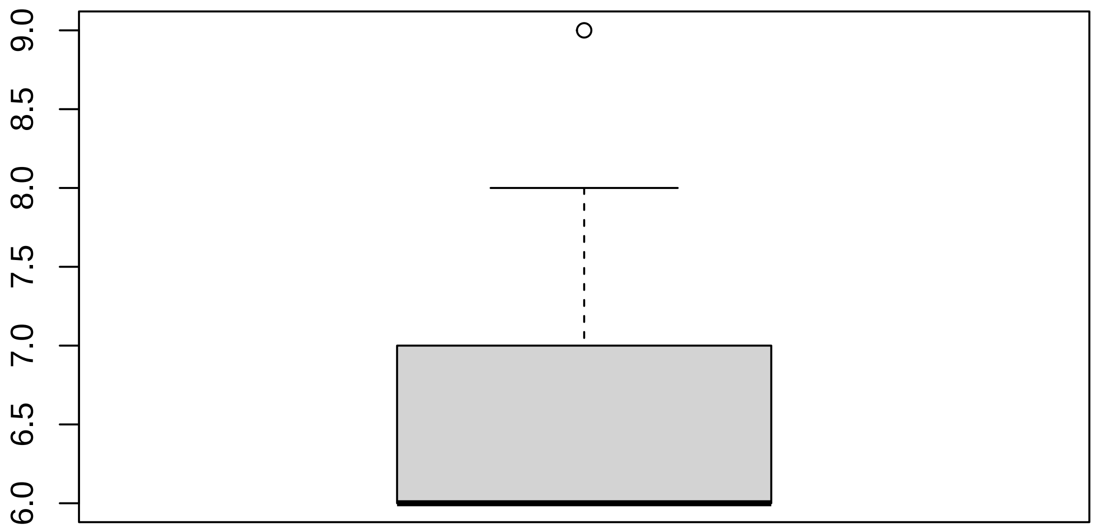


```
##
##  Shapiro-Wilk normality test
##
## data:  x
## W = 0.6447, p-value = 1.011e-06
##
##
##  One-sample Kolmogorov-Smirnov test
##
## data:  x
## D = 0.41645, p-value = 0.0002423
## alternative hypothesis: two-sided
```

```
normality(Data$`Lymphocytes CD4 Count`)
```

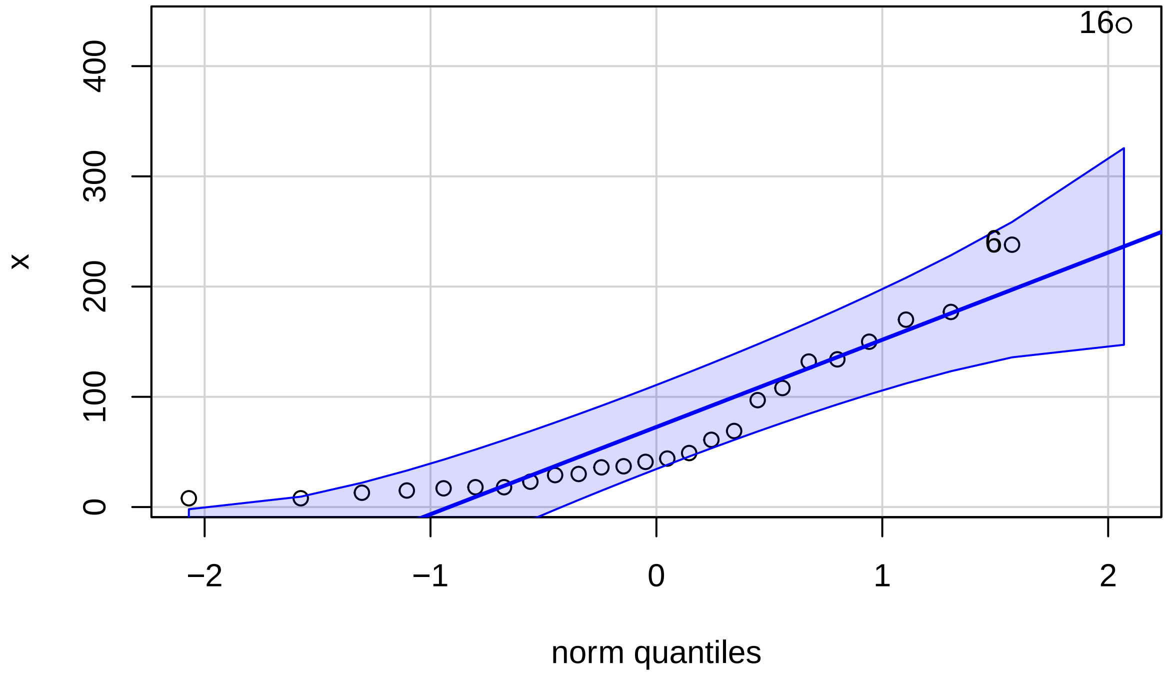


```
## Warning in stats::ks.test(x, y, ..., alternative = alternative, exact = exact):
## ties should not be present for the Kolmogorov-Smirnov test
```

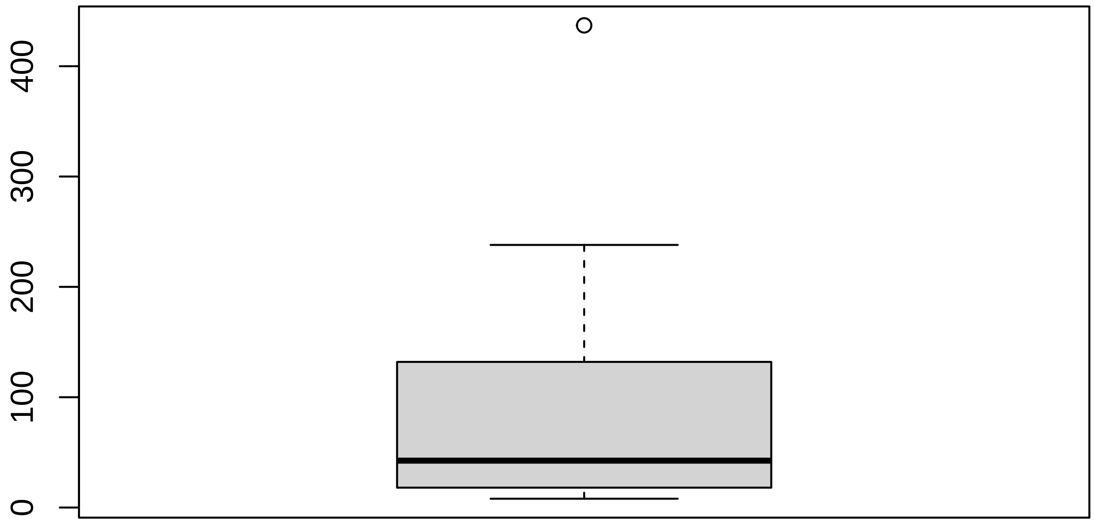


```
##
##  Shapiro-Wilk normality test
##
## data:  x
## W = 0.7376, p-value = 1.814e-05
##
##
##  One-sample Kolmogorov-Smirnov test
##
## data:  x
## D = 0.21621, p-value = 0.1758
## alternative hypothesis: two-sided
```

```
normality(Data$`Viral Loads`)
```

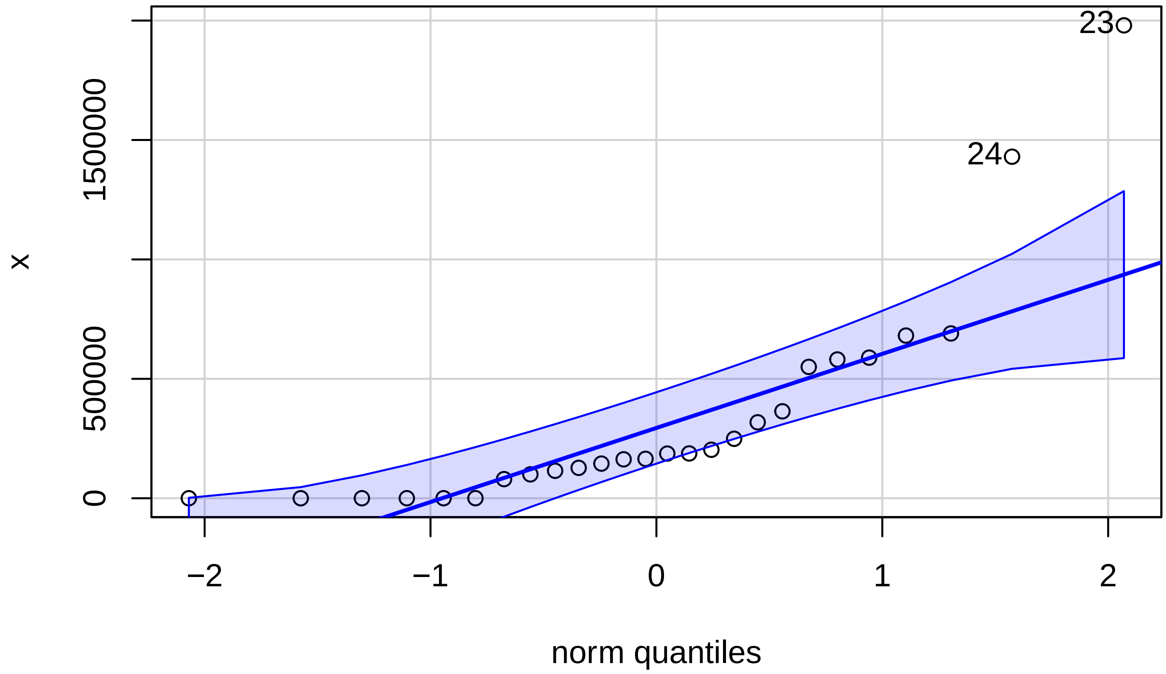

x
0
500000
1500000
−2
−1
0
1
2
norm quantiles
23
24

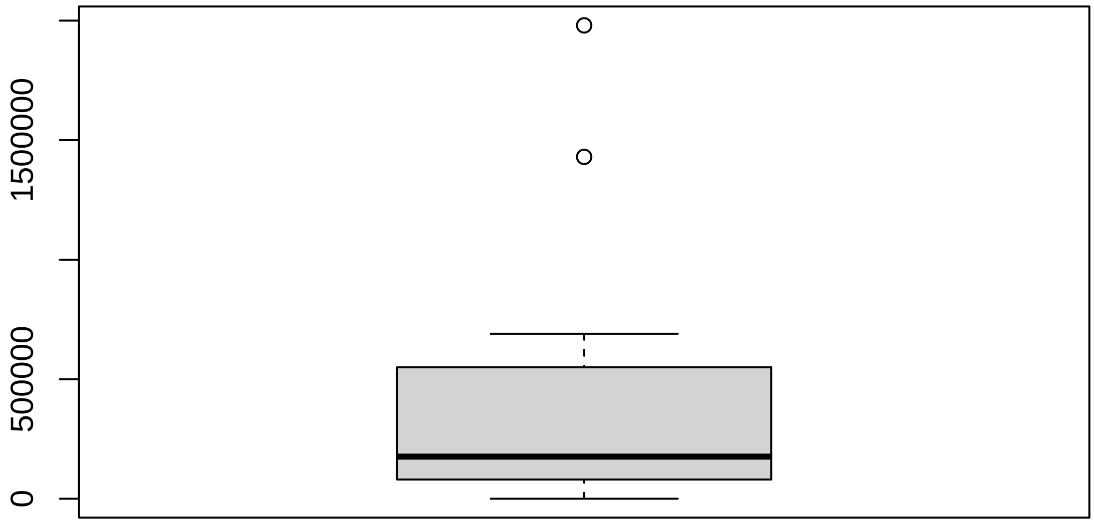


```
##
##  Shapiro-Wilk normality test
##
## data:  x
## W = 0.70635, p-value = 6.485e-06
##
##
##  One-sample Kolmogorov-Smirnov test
##
## data:  x
## D = 0.2338, p-value = 0.09848
## alternative hypothesis: two-sided
```

```
norm.analysis <- function(x, y){
  b <- car::leveneTest(x~y)
  c <- t.test(x ~ y, paired = F)
  d <- t.test(x ~ y, paired = F, var.equal=T)
  e <- FSA::Summarize(x~y)
  cat("\nDescriptivos:\n")
  print(e)
  cat("\nPreuba de Levene:\n")
  print(b)
  cat("\nTestWlech:\n")
  print(c)
  cat("\nIguales:\n")
```

```
  print(d)
  boxplot(x~y)
}
```

```
norm.analysis(Data$Age, Data$Groups)
```

```
##
## Descriptivos:
##         y  n     mean       sd min    Q1 median    Q3 max
## 1 M.Bovis 12 38.83333 8.860878  25 30.75     39 43.75  56
## 2    M.TB 14 41.21429 9.258498  26 33.50     43 48.75  54
##
## Preuba de Levene:
## Levene's Test for Homogeneity of Variance (center = median)
##       Df F value Pr(>F)
## group  1  0.2291 0.6365
##       24
##
## TestWlech:
##
##  Welch Two Sample t-test
##
## data:  x by y
## t = -0.66901, df = 23.676, p-value = 0.51
## alternative hypothesis: true difference in means between group M.Bovis and group M.TB is not equal t
## 95 percent confidence interval:
##  -9.731478  4.969573
## sample estimates:
## mean in group M.Bovis    mean in group M.TB
##              38.83333              41.21429
##
##
## Iguales:
##
##  Two Sample t-test
##
## data:  x by y
## t = -0.66667, df = 24, p-value = 0.5113
## alternative hypothesis: true difference in means between group M.Bovis and group M.TB is not equal t
## 95 percent confidence interval:
##  -9.752022  4.990117
## sample estimates:
## mean in group M.Bovis    mean in group M.TB
##              38.83333              41.21429
```

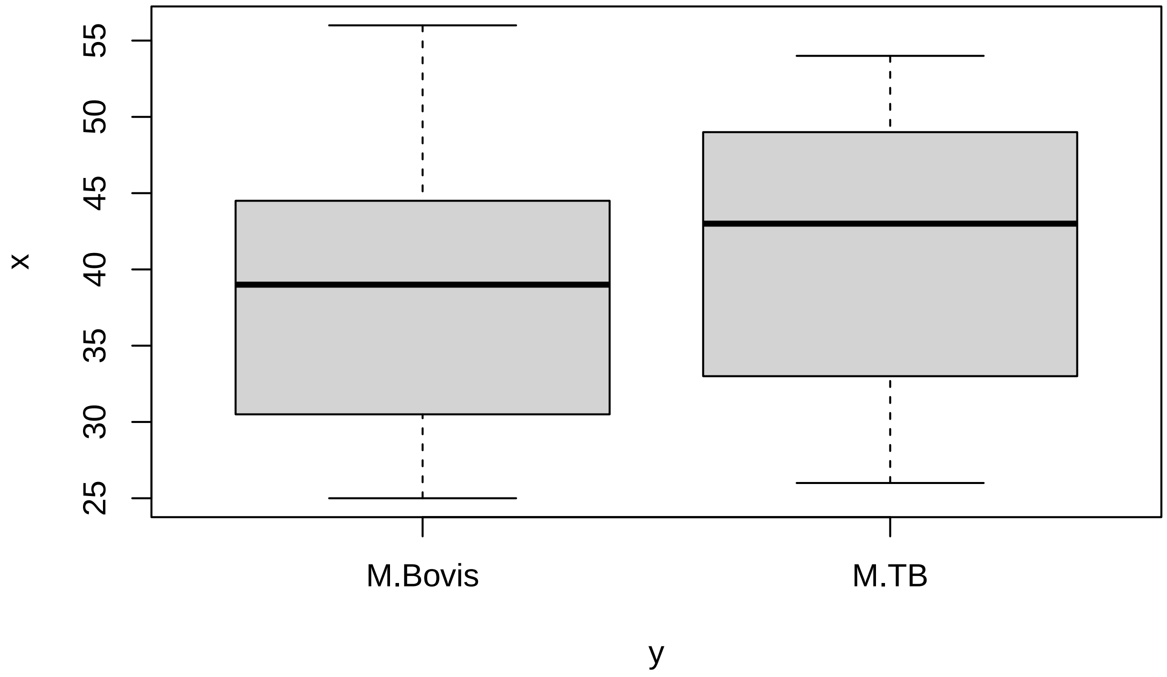


```
no.norm.analysis <- function(x, y){
  a <- FSA::Summarize(x ~ y)
  b <- wilcox.test(x ~ y, paired = F)
  cat("\nDescriptivos:\n")
  print(a)
  cat("\nU Mann Whitney:\n")
  print(b)
  boxplot(x~y)
}
```

```
no.norm.analysis(Data$Charlson, Data$Groups)
```

```
## Warning in wilcox.test.default(x = c(6, 6, 8, 8, 7, 6, 6, 6, 6, 6, 6, 6), :
## cannot compute exact p-value with ties
```

```
##
## Descriptivos:
##         y  n     mean        sd min Q1 median   Q3 max
## 1 M.Bovis 12 6.416667 0.7929615   6  6      6 6.25   8
## 2    M.TB 14 6.642857 1.0082081   6  6      6 7.00   9
##
## U Mann Whitney:
##
##  Wilcoxon rank sum test with continuity correction
##
```

```
## data:  x by y
## W = 74.5, p-value = 0.57
## alternative hypothesis: true location shift is not equal to 0
```

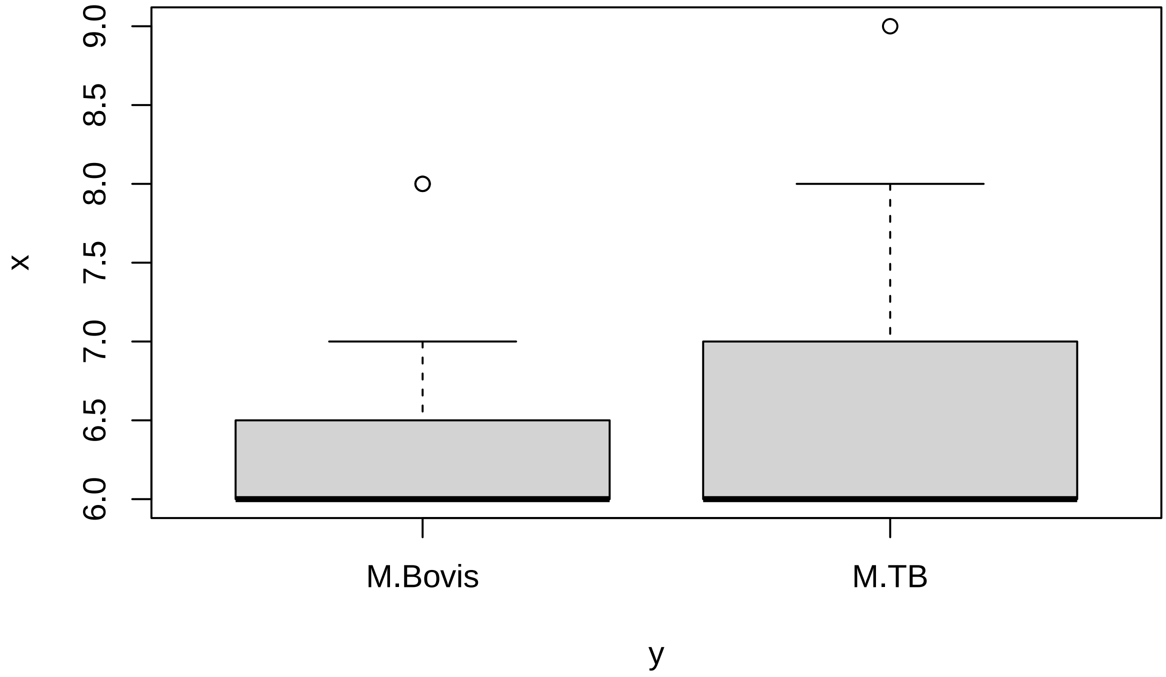


```
no.norm.analysis(Data$`Lymphocytes CD4 Count`, Data$Groups)
```

```
## Warning in wilcox.test.default(x = c(177, 108, 97, 150, 44, 238, 170, 30, :
## cannot compute exact p-value with ties
```

```
##
## Descriptivos:
##          y  n      mean        sd min    Q1 median  Q3 max
## 1 M.Bovis 12 105.00000  69.87782  17 42.00  102.5 155 238
## 2    M.TB 14  64.21429 112.30054   8 15.75   26.0  47 437
##
## U Mann Whitney:
##
##  Wilcoxon rank sum test with continuity correction
##
## data:  x by y
## W = 129, p-value = 0.02204
## alternative hypothesis: true location shift is not equal to 0
```

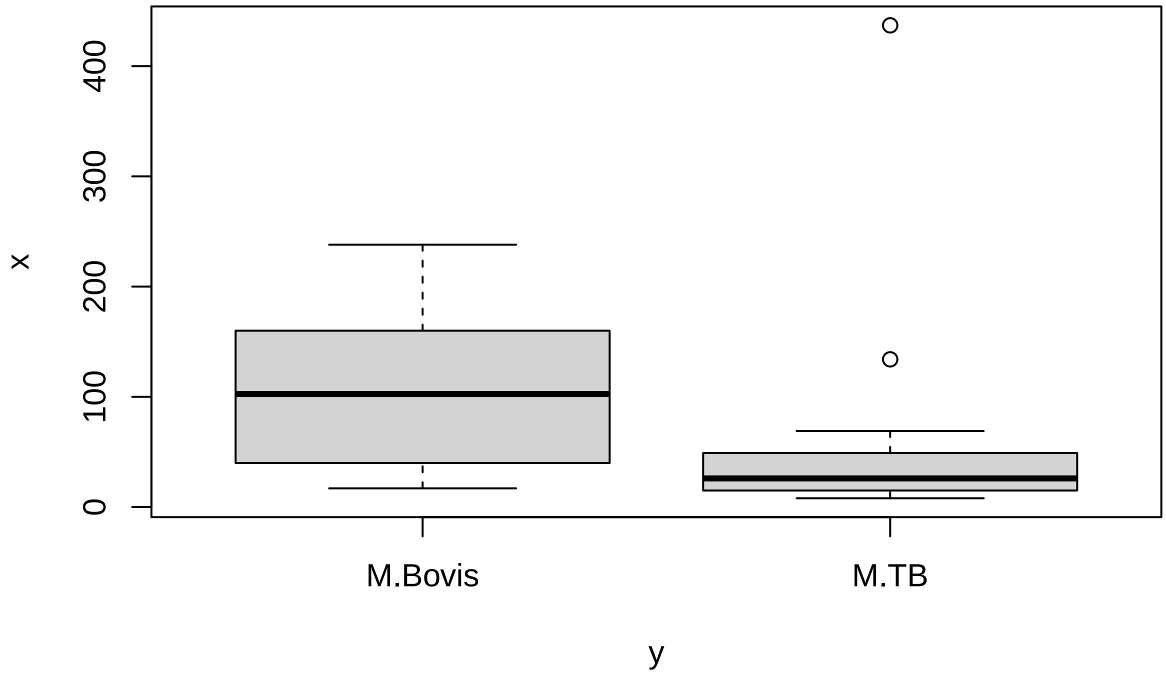


```
no.norm.analysis(Data$`Viral Loads`, Data$Groups)
```

```
##
## Descriptivos:
##          y  n     mean       sd min       Q1   median       Q3     max
## 1 M.Bovis 12 198072.5 204092.5  19    277.0 176260.0 266250.0  588673
## 2    M.TB 14 466321.9 584811.0  20 106826.2 175009.5 656014.2 1980000
##
## U Mann Whitney:
##
##  Wilcoxon rank sum exact test
##
## data:  x by y
## W = 68, p-value = 0.4319
## alternative hypothesis: true location shift is not equal to 0
```

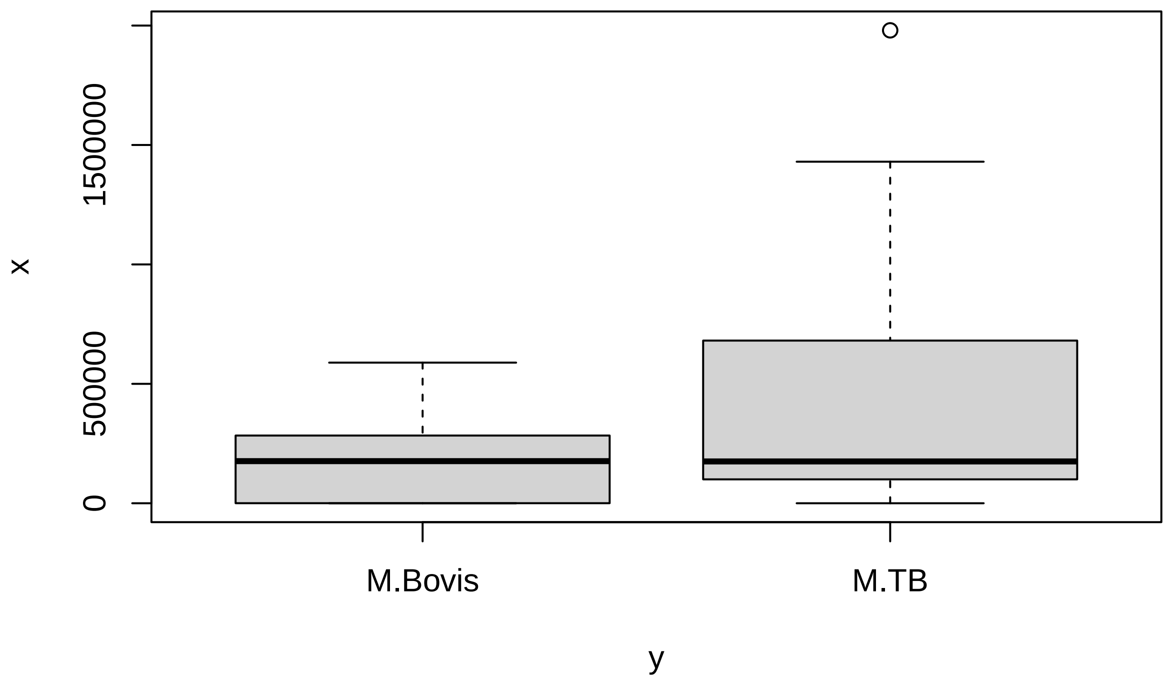
x
0
500000
1500000
M.Bovis
M.TB
y

Clinical, imaging and microbiological characteristics of patients with M tuberculosis or M. bovis infection in HIV-infected subjects treated at the HCGFAA.

Andrea Torres Rojas

2025-06-01

```
library(readxl)
Data2 <- read_excel("C:/Users/andre/OneDrive/Escritorio/Data2.xlsx")
head(Data2)
```

```
## # A tibble: 6 x 34
##   Groups    ID Fever Cough `Weight loss` Lymphadenopathies `Stomach ache`
##    <dbl> <dbl> <dbl> <dbl>         <dbl>             <dbl>          <dbl>
## 1      1     1     1     2             1                 1              1
## 2      1     2     1     1             1                 2              1
## 3      1     3     2     2             2                 1              2
## 4      1     4     1     1             1                 1              1
## 5      1     5     1     2             2                 1              1
## 6      1     6     1     2             2                 1              1
## # ... with 27 more variables: Neurologic <dbl>, Pulmonary <dbl>,
## #   Extrapulmonary <dbl>, Pulmonary and extrapulmonary <dbl>,
## #   Miliar pattern <dbl>, Cavitations <dbl>, Nodular <dbl>,
## #   Pleural effusion <dbl>, Bronchiectasis <dbl>,
## #   Retroperitoneal lymphadenopathy <dbl>, Collections in psoas <dbl>,
## #   Hepatomegaly <dbl>, Splenomegaly <dbl>, Splenic abscesses <dbl>,
## #   IS Pulmonary <dbl>, IS Ganglionar <dbl>, IS Abdominal <dbl>, ...
```

```
Data2$Groups<-as.factor(Data2$Groups)
Data2$Groups<-factor(Data2$Groups,levels = c(1,2), labels = c("M.Bovis", "M.TB"))
Data2$Fever<-as.factor(Data2$Fever)
Data2$Fever<-factor(Data2$Fever, levels = c(1,2), labels = c("yes", "no"))
Data2$Cough<-as.factor(Data2$Cough)
Data2$Cough<-factor(Data2$Cough, levels = c(1,2), labels = c("yes", "no"))
Data2$`Weight loss`<-as.factor(Data2$`Weight loss`)
Data2$`Weight loss`<-factor(Data2$`Weight loss`, levels = c(1,2), labels = c("yes", "no"))
Data2$Lymphadenopathies<-as.factor(Data2$Lymphadenopathies)
Data2$Lymphadenopathies<-factor(Data2$Lymphadenopathies, levels = c(1,2), labels = c("yes", "no"))
Data2$`Stomach ache`<-as.factor(Data2$`Stomach ache`)
Data2$`Stomach ache`<-factor(Data2$`Stomach ache`, levels = c(1,2), labels = c("yes", "no"))
Data2$Neurologic<-as.factor(Data2$Neurologic)
Data2$Neurologic<-factor(Data2$Neurologic, levels = c(1,2), labels = c("yes", "no"))
Data2$Pulmonary<-as.factor(Data2$Pulmonary)
Data2$Pulmonary<-factor(Data2$Pulmonary, levels = c(1,2), labels = c("yes", "no"))
```

```r
Data2$Extrapulmonary<-as.factor(Data2$Extrapulmonary)
Data2$Extrapulmonary<-factor(Data2$Extrapulmonary, levels = c(1,2), labels = c("yes", "no"))
Data2$`Pulmonary and extrapulmonary`<-as.factor(Data2$`Pulmonary and extrapulmonary`)
Data2$`Pulmonary and extrapulmonary`<-factor(Data2$`Pulmonary and extrapulmonary`, levels = c(1,2), labe
Data2$`Miliar pattern`<-as.factor(Data2$`Miliar pattern`)
Data2$`Miliar pattern`<-factor(Data2$`Miliar pattern`, levels = c(1,2), labels = c("yes", "no"))
Data2$Cavitations<-as.factor(Data2$Cavitations)
Data2$Cavitations<-factor(Data2$Cavitations, levels = c(1,2), labels = c("yes", "no"))
Data2$Nodular<-as.factor(Data2$Nodular)
Data2$Nodular<-factor(Data2$Nodular, levels = c(1,2), labels = c("yes", "no"))
Data2$`Pleural effusion`<-as.factor(Data2$`Pleural effusion`)
Data2$`Pleural effusion`<-factor(Data2$`Pleural effusion`, levels = c(1,2), labels = c("yes", "no"))
Data2$Bronchiectasis<-as.factor(Data2$Bronchiectasis)
Data2$Bronchiectasis<-factor(Data2$Bronchiectasis, levels = c(1,2), labels = c("yes", "no"))
Data2$`Retroperitoneal lymphadenopathy`<-as.factor(Data2$`Retroperitoneal lymphadenopathy`)
Data2$`Retroperitoneal lymphadenopathy`<-factor(Data2$`Retroperitoneal lymphadenopathy`, levels = c(1,2
Data2$`Collections in psoas`<-as.factor(Data2$`Collections in psoas`)
Data2$`Collections in psoas`<-factor(Data2$`Collections in psoas`, levels = c(1,2), labels = c("yes", "
Data2$Hepatomegaly<-as.factor(Data2$Hepatomegaly)
Data2$Hepatomegaly<-factor(Data2$Hepatomegaly, levels = c(1,2), labels = c("yes", "no"))
Data2$Splenomegaly<-as.factor(Data2$Splenomegaly)
Data2$Splenomegaly<-factor(Data2$Splenomegaly, levels = c(1,2), labels = c("yes", "no"))
Data2$`Splenic abscesses`<-as.factor(Data2$`Splenic abscesses`)
Data2$`Splenic abscesses`<-factor(Data2$`Splenic abscesses`, levels = c(1,2), labels = c("yes", "no"))
Data2$`IS Pulmonary`<-as.factor(Data2$`IS Pulmonary`)
Data2$`IS Pulmonary`<-factor(Data2$`IS Pulmonary`, levels = c(1,2), labels = c("yes", "no"))
Data2$`IS Ganglionar`<-as.factor(Data2$`IS Ganglionar`)
Data2$`IS Ganglionar`<-factor(Data2$`IS Ganglionar`, levels = c(1,2), labels = c("yes", "no"))
Data2$`IS Abdominal`<-as.factor(Data2$`IS Abdominal`)
Data2$`IS Abdominal`<-factor(Data2$`IS Abdominal`, levels = c(1,2), labels = c("yes", "no"))
Data2$`IS CSF`<-as.factor(Data2$`IS CSF`)
Data2$`IS CSF`<-factor(Data2$`IS CSF`, levels = c(1,2), labels = c("yes", "no"))
Data2$`IS Psoas`<-as.factor(Data2$`IS Psoas`)
Data2$`IS Psoas`<-factor(Data2$`IS Psoas`, levels = c(1,2), labels = c("yes", "no"))
Data2$`IS Genitourinary`<-as.factor(Data2$`IS Genitourinary`)
Data2$`IS Genitourinary`<-factor(Data2$`IS Genitourinary`, levels = c(1,2), labels = c("yes", "no"))
Data2$`IS Bones`<-as.factor(Data2$`IS Bones`)
Data2$`IS Bones`<-factor(Data2$`IS Bones`, levels = c(1,2), labels = c("yes", "no"))
Data2$`Xpert MTb/RIF`<-as.factor(Data2$`Xpert MTb/RIF`)
Data2$`Xpert MTb/RIF`<-factor(Data2$`Xpert MTb/RIF`, levels = c(1,2), labels = c("yes", "no"))
Data2$`Resistance to rifampicin by Xpert MTb/RIF`<-as.factor(Data2$`Resistance to rifampicin by Xpert MT
Data2$`Resistance to rifampicin by Xpert MTb/RIF`<-factor(Data2$`Resistance to rifampicin by Xpert MTb/R
Data2$Tinction<-as.factor(Data2$Tinction)
Data2$Tinction<-factor(Data2$Tinction, levels = c(1,2), labels = c("yes", "no"))
Data2$Histopathology<-as.factor(Data2$Histopathology)
Data2$Histopathology<-factor(Data2$Histopathology, levels = c(1,2), labels = c("yes", "no"))
```

```r
str(Data2)
```

```
## tibble [26 x 34] (S3: tbl_df/tbl/data.frame)
##  $ Groups                                   : Factor w/ 2 levels "M.Bovis","M.TB": 1 1 1 1 1 1 1 1 1
##  $ ID                                       : num [1:26] 1 2 3 4 5 6 7 8 9 10 ...
##  $ Fever                                    : Factor w/ 2 levels "yes","no": 1 1 2 1 1 1 1 1 1 1 ...
```

```
##  $ Cough                                    : Factor w/ 2 levels "yes","no": 2 1 2 1 2 2 1 1 1 1 ...
##  $ Weight loss                              : Factor w/ 2 levels "yes","no": 1 1 2 1 2 2 2 2 1 1 ...
##  $ Lymphadenopathies                        : Factor w/ 2 levels "yes","no": 1 2 1 1 1 1 1 1 1 1 ...
##  $ Stomach ache                             : Factor w/ 2 levels "yes","no": 1 1 2 1 1 1 2 2 1 1 ...
##  $ Neurologic                               : Factor w/ 2 levels "yes","no": 2 2 2 1 2 2 2 2 2 2 ...
##  $ Pulmonary                                : Factor w/ 2 levels "yes","no": 2 2 2 2 1 2 2 2 2 2 ...
##  $ Extrapulmonary                           : Factor w/ 2 levels "yes","no": 2 2 1 1 2 1 2 2 1 2 ...
##  $ Pulmonary and extrapulmonary             : Factor w/ 2 levels "yes","no": 1 1 1 2 1 1 1 1 2 1 ...
##  $ Miliar pattern                           : Factor w/ 2 levels "yes","no": 2 1 2 2 2 1 2 1 2 2 ...
##  $ Cavitations                              : Factor w/ 2 levels "yes","no": 2 2 2 2 2 2 1 2 2 2 ...
##  $ Nodular                                  : Factor w/ 2 levels "yes","no": 2 2 2 2 2 2 2 2 2 2 ...
##  $ Pleural effusion                         : Factor w/ 2 levels "yes","no": 2 2 2 2 2 2 2 2 2 1 ...
##  $ Bronchiectasis                           : Factor w/ 2 levels "yes","no": 2 2 2 2 2 1 2 2 2 2 ...
##  $ Retroperitoneal lymphadenopathy          : Factor w/ 2 levels "yes","no": 1 1 1 1 1 1 2 1 1 1 ...
##  $ Collections in psoas                     : Factor w/ 2 levels "yes","no": 2 1 2 2 1 1 1 2 2 2 ...
##  $ Hepatomegaly                             : Factor w/ 2 levels "yes","no": 1 1 1 2 1 2 1 2 2 1 ...
##  $ Splenomegaly                             : Factor w/ 2 levels "yes","no": 2 1 1 1 1 1 1 2 2 1 ...
##  $ Splenic abscesses                        : Factor w/ 2 levels "yes","no": 2 1 2 1 2 1 2 2 1 1 ...
##  $ IS Pulmonary                             : Factor w/ 2 levels "yes","no": 2 2 2 2 1 2 2 2 2 2 ...
##  $ IS Ganglionar                            : Factor w/ 2 levels "yes","no": 2 1 1 2 1 2 2 1 2 1 ...
##  $ IS Abdominal                             : Factor w/ 2 levels "yes","no": 2 2 1 2 1 2 2 2 2 2 ...
##  $ IS CSF                                   : Factor w/ 2 levels "yes","no": 2 2 2 2 2 2 2 2 2 2 ...
##  $ IS Psoas                                 : Factor w/ 2 levels "yes","no": 2 2 2 2 1 2 2 2 2 2 ...
##  $ IS Genitourinary                         : Factor w/ 2 levels "yes","no": 2 2 2 2 2 2 2 2 2 2 ...
##  $ IS Bones                                 : Factor w/ 2 levels "yes","no": 2 2 1 2 2 2 1 2 2 2 ...
##  $ Tinction                                 : Factor w/ 2 levels "yes","no": 1 1 1 1 1 1 1 2 2 1 ...
##  $ Xpert MTb/RIF                            : Factor w/ 2 levels "yes","no": 1 1 1 1 1 2 1 1 1 1 ...
##  $ Resistance to rifampicin by Xpert MTb/RIF: Factor w/ 2 levels "yes","no": 2 2 2 2 2 2 2 2 2 2 ...
##  $ Crops grown                              : num [1:26] 1 5 6 0 15 4 5 1 0 1 ...
##  $ Positive growns                          : num [1:26] 1 0 0 0 0 0 0 0 0 1 ...
##  $ Histopathology                           : Factor w/ 2 levels "yes","no": 1 2 1 2 2 1 2 2 2 2 ...
```

```
Table.Frequencies <- function(x){
  y <- table(x)
  data_freq <-
    data.frame(Niveles= names(y),
               Freq =as.numeric(y),
               Freq_Rela = as.numeric(prop.table(y)),
               Freq_Acum = as.numeric(cumsum(y)),
               Freq_Rela_Acum = as.numeric (cumsum(prop.table(y))))

  data_freq}
```

```
table(Data2$Fever, Data2$Groups)
```

```
##
##       M.Bovis M.TB
##   yes      10    8
##   no        2    6
```

```
x<-table(Data2$Fever, Data2$Groups)
fisher.test(x)
```

```
##
##  Fisher's Exact Test for Count Data
##
## data:  x
## p-value = 0.2164
## alternative hypothesis: true odds ratio is not equal to 1
## 95 percent confidence interval:
##   0.4642738 45.5783438
## sample estimates:
## odds ratio
##   3.563294
```

```
gr <- split(Data2$Fever, Data2$Groups)
results <- lapply(gr, Table.Frequencies)
data.frame(results)
```

```
##   M.Bovis.Niveles M.Bovis.Freq M.Bovis.Freq_Rela M.Bovis.Freq_Acum
## 1             yes           10         0.8333333                10
## 2              no            2         0.1666667                12
##   M.Bovis.Freq_Rela_Acum M.TB.Niveles M.TB.Freq M.TB.Freq_Rela M.TB.Freq_Acum
## 1              0.8333333          yes         8      0.5714286              8
## 2              1.0000000           no         6      0.4285714             14
##   M.TB.Freq_Rela_Acum
## 1           0.5714286
## 2           1.0000000
```

```
table(Data2$Cough, Data2$Groups)
```

```
##
##       M.Bovis M.TB
##   yes       8    6
##   no        4    8
```

```
x<-table(Data2$Cough, Data2$Groups)
fisher.test(x)
```

```
##
##  Fisher's Exact Test for Count Data
##
## data:  x
## p-value = 0.2671
## alternative hypothesis: true odds ratio is not equal to 1
## 95 percent confidence interval:
##   0.4223446 17.9834426
## sample estimates:
## odds ratio
##   2.564529
```

```
gr <- split(Data2$Cough, Data2$Groups)
results <- lapply(gr, Table.Frequencies)
data.frame(results)
```

```
##   M.Bovis.Niveles M.Bovis.Freq M.Bovis.Freq_Rela M.Bovis.Freq_Acum
## 1             yes            8         0.6666667                 8
## 2              no            4         0.3333333                12
##   M.Bovis.Freq_Rela_Acum M.TB.Niveles M.TB.Freq M.TB.Freq_Rela M.TB.Freq_Acum
## 1              0.6666667          yes         6      0.4285714              6
## 2              1.0000000           no         8      0.5714286             14
##   M.TB.Freq_Rela_Acum
## 1           0.4285714
## 2           1.0000000
```

```
table(Data2$`Weight loss`, Data2$Groups)
```

```
##
##       M.Bovis M.TB
##   yes       7   11
##   no        5    3
```

```
x<-table(Data2$`Weight loss`, Data2$Groups)
fisher.test(x)
```

```
##
##  Fisher's Exact Test for Count Data
##
## data:  x
## p-value = 0.4009
## alternative hypothesis: true odds ratio is not equal to 1
## 95 percent confidence interval:
##  0.0459793 2.8181497
## sample estimates:
## odds ratio
##  0.3967424
```

```
gr <- split(Data2$`Weight loss`, Data2$Groups)
results <- lapply(gr, Table.Frequencies)
data.frame(results)
```

```
##   M.Bovis.Niveles M.Bovis.Freq M.Bovis.Freq_Rela M.Bovis.Freq_Acum
## 1             yes            7         0.5833333                 7
## 2              no            5         0.4166667                12
##   M.Bovis.Freq_Rela_Acum M.TB.Niveles M.TB.Freq M.TB.Freq_Rela M.TB.Freq_Acum
## 1              0.5833333          yes        11      0.7857143             11
## 2              1.0000000           no         3      0.2142857             14
##   M.TB.Freq_Rela_Acum
## 1           0.7857143
## 2           1.0000000
```

```
table(Data2$Lymphadenopathies, Data2$Groups)
```

```
##
##       M.Bovis M.TB
##   yes      11   10
##   no        1    4
```

```r
x<-table(Data2$Lymphadenopathies, Data2$Groups)
fisher.test(x)
```

```
##
##  Fisher's Exact Test for Count Data
##
## data:  x
## p-value = 0.3304
## alternative hypothesis: true odds ratio is not equal to 1
## 95 percent confidence interval:
##    0.3357131 235.6498533
## sample estimates:
## odds ratio
##   4.172678
```

```r
gr <- split(Data2$Lymphadenopathies, Data2$Groups)
results <- lapply(gr, Table.Frequencies)
data.frame(results)
```

```
##   M.Bovis.Niveles M.Bovis.Freq M.Bovis.Freq_Rela M.Bovis.Freq_Acum
## 1             yes           11        0.91666667                11
## 2              no            1        0.08333333                12
##   M.Bovis.Freq_Rela_Acum M.TB.Niveles M.TB.Freq M.TB.Freq_Rela M.TB.Freq_Acum
## 1              0.9166667          yes        10      0.7142857             10
## 2              1.0000000           no         4      0.2857143             14
##   M.TB.Freq_Rela_Acum
## 1           0.7142857
## 2           1.0000000
```

```r
table(Data2$`Stomach ache`, Data2$Groups)
```

```
##
##       M.Bovis M.TB
##   yes       8   12
##   no        4    2
```

```r
x<-table(Data2$`Stomach ache`, Data2$Groups)
fisher.test(x)
```

```
##
##  Fisher's Exact Test for Count Data
##
## data:  x
## p-value = 0.3652
## alternative hypothesis: true odds ratio is not equal to 1
## 95 percent confidence interval:
##  0.02563046 3.11485520
## sample estimates:
## odds ratio
##  0.3481014
```

```
gr <- split(Data2$`Stomach ache`, Data2$Groups)
results <- lapply(gr, Table.Frequencies)
data.frame(results)
```

```
##   M.Bovis.Niveles M.Bovis.Freq M.Bovis.Freq_Rela M.Bovis.Freq_Acum
## 1             yes            8         0.6666667                 8
## 2              no            4         0.3333333                12
##   M.Bovis.Freq_Rela_Acum M.TB.Niveles M.TB.Freq M.TB.Freq_Rela M.TB.Freq_Acum
## 1              0.6666667          yes        12      0.8571429             12
## 2              1.0000000           no         2      0.1428571             14
##   M.TB.Freq_Rela_Acum
## 1           0.8571429
## 2           1.0000000
```

```
table(Data2$Pulmonary, Data2$Groups)
```

```
##
##       M.Bovis M.TB
##   yes       1    8
##   no       11    6
```

```
x<-table(Data2$Pulmonary, Data2$Groups)
fisher.test(x)
```

```
##
##  Fisher's Exact Test for Count Data
##
## data:  x
## p-value = 0.01446
## alternative hypothesis: true odds ratio is not equal to 1
## 95 percent confidence interval:
##  0.001418488 0.788785489
## sample estimates:
## odds ratio
##  0.0760998
```

```
gr <- split(Data2$Pulmonary, Data2$Groups)
results <- lapply(gr, Table.Frequencies)
data.frame(results)
```

```
##   M.Bovis.Niveles M.Bovis.Freq M.Bovis.Freq_Rela M.Bovis.Freq_Acum
## 1             yes            1        0.08333333                 1
## 2              no           11        0.91666667                12
##   M.Bovis.Freq_Rela_Acum M.TB.Niveles M.TB.Freq M.TB.Freq_Rela M.TB.Freq_Acum
## 1             0.08333333          yes         8      0.5714286              8
## 2             1.00000000           no         6      0.4285714             14
##   M.TB.Freq_Rela_Acum
## 1           0.5714286
## 2           1.0000000
```

```
table(Data2$Extrapulmonary, Data2$Groups)
```

```
##
##       M.Bovis M.TB
##   yes       4    3
##   no        8   11
```

```
x<-table(Data2$Extrapulmonary, Data2$Groups)
fisher.test(x)
```

```
##
##  Fisher's Exact Test for Count Data
##
## data:  x
## p-value = 0.6652
## alternative hypothesis: true odds ratio is not equal to 1
## 95 percent confidence interval:
##   0.2293539 15.8859832
## sample estimates:
## odds ratio
##   1.790451
```

```
gr <- split(Data2$Extrapulmonary, Data2$Groups)
results <- lapply(gr, Table.Frequencies)
data.frame(results)
```

```
##   M.Bovis.Niveles M.Bovis.Freq M.Bovis.Freq_Rela M.Bovis.Freq_Acum
## 1             yes            4         0.3333333                 4
## 2              no            8         0.6666667                12
##   M.Bovis.Freq_Rela_Acum M.TB.Niveles M.TB.Freq M.TB.Freq_Rela M.TB.Freq_Acum
## 1              0.3333333          yes         3      0.2142857              3
## 2              1.0000000           no        11      0.7857143             14
##   M.TB.Freq_Rela_Acum
## 1           0.2142857
## 2           1.0000000
```

```
table(Data2$`Pulmonary and extrapulmonary`, Data2$Groups)
```

```
##
##       M.Bovis M.TB
##   yes       9    2
##   no        3   12
```

```
x<-table(Data2$`Pulmonary and extrapulmonary`, Data2$Groups)
fisher.test(x)
```

```
##
##  Fisher's Exact Test for Count Data
##
## data:  x
```

```
## p-value = 0.004314
## alternative hypothesis: true odds ratio is not equal to 1
## 95 percent confidence interval:
##    1.899944 224.290998
## sample estimates:
## odds ratio
##   15.44496
```

```
gr <- split(Data2$`Pulmonary and extrapulmonary`, Data2$Groups)
results <- lapply(gr, Table.Frequencies)
data.frame(results)
```

```
##   M.Bovis.Niveles M.Bovis.Freq M.Bovis.Freq_Rela M.Bovis.Freq_Acum
## 1             yes            9              0.75                 9
## 2              no            3              0.25                12
##   M.Bovis.Freq_Rela_Acum M.TB.Niveles M.TB.Freq M.TB.Freq_Rela M.TB.Freq_Acum
## 1                   0.75          yes         2      0.1428571              2
## 2                   1.00           no        12      0.8571429             14
##   M.TB.Freq_Rela_Acum
## 1           0.1428571
## 2           1.0000000
```

```
table(Data2$`Miliar pattern`, Data2$Groups)
```

```
##
##       M.Bovis M.TB
##   yes       4    5
##   no        8    9
```

```
x<-table(Data2$`Miliar pattern`, Data2$Groups)
fisher.test(x)
```

```
##
##  Fisher's Exact Test for Count Data
##
## data:  x
## p-value = 1
## alternative hypothesis: true odds ratio is not equal to 1
## 95 percent confidence interval:
##  0.1288941 6.0041037
## sample estimates:
## odds ratio
##  0.9036515
```

```
gr <- split(Data2$`Miliar pattern`, Data2$Groups)
results <- lapply(gr, Table.Frequencies)
data.frame(results)
```

```
##   M.Bovis.Niveles M.Bovis.Freq M.Bovis.Freq_Rela M.Bovis.Freq_Acum
## 1             yes            4         0.3333333                 4
## 2              no            8         0.6666667                12
```

```
##   M.Bovis.Freq_Rela_Acum M.TB.Niveles M.TB.Freq M.TB.Freq_Rela M.TB.Freq_Acum
## 1              0.3333333          yes         5      0.3571429              5
## 2              1.0000000           no         9      0.6428571             14
##   M.TB.Freq_Rela_Acum
## 1           0.3571429
## 2           1.0000000
```

```
table(Data2$Cavitations, Data2$Groups)
```

```
##
##       M.Bovis M.TB
##   yes       1    3
##   no       11   11
```

```
x<-table(Data2$Cavitations, Data2$Groups)
fisher.test(x)
```

```
##
##  Fisher's Exact Test for Count Data
##
## data:  x
## p-value = 0.5983
## alternative hypothesis: true odds ratio is not equal to 1
## 95 percent confidence interval:
##  0.005835461 5.120541458
## sample estimates:
## odds ratio
##  0.3468369
```

```
gr <- split(Data2$Cavitations, Data2$Groups)
results <- lapply(gr, Table.Frequencies)
data.frame(results)
```

```
##   M.Bovis.Niveles M.Bovis.Freq M.Bovis.Freq_Rela M.Bovis.Freq_Acum
## 1             yes            1        0.08333333                 1
## 2              no           11        0.91666667                12
##   M.Bovis.Freq_Rela_Acum M.TB.Niveles M.TB.Freq M.TB.Freq_Rela M.TB.Freq_Acum
## 1             0.08333333          yes         3      0.2142857              3
## 2             1.00000000           no        11      0.7857143             14
##   M.TB.Freq_Rela_Acum
## 1           0.2142857
## 2           1.0000000
```

```
table(Data2$Nodular, Data2$Groups)
```

```
##
##       M.Bovis M.TB
##   yes       0    0
##   no       12   14
```

```
x<-table(Data2$Nodular, Data2$Groups)
fisher.test(x)
```

```
##
##  Fisher's Exact Test for Count Data
##
## data:  x
## p-value = 1
## alternative hypothesis: true odds ratio is not equal to 1
## 95 percent confidence interval:
##    0 Inf
## sample estimates:
## odds ratio
##          0
```

```
gr <- split(Data2$Nodular, Data2$Groups)
results <- lapply(gr, Table.Frequencies)
data.frame(results)
```

```
##   M.Bovis.Niveles M.Bovis.Freq M.Bovis.Freq_Rela M.Bovis.Freq_Acum
## 1             yes            0                 0                 0
## 2              no           12                 1                12
##   M.Bovis.Freq_Rela_Acum M.TB.Niveles M.TB.Freq M.TB.Freq_Rela M.TB.Freq_Acum
## 1                      0          yes         0              0              0
## 2                      1           no        14              1             14
##   M.TB.Freq_Rela_Acum
## 1                   0
## 2                   1
```

```
table(Data2$`Pleural effusion`, Data2$Groups)
```

```
##
##       M.Bovis M.TB
##   yes       2    0
##   no       10   14
```

```
x<-table(Data2$`Pleural effusion`, Data2$Groups)
fisher.test(x)
```

```
##
##  Fisher's Exact Test for Count Data
##
## data:  x
## p-value = 0.2031
## alternative hypothesis: true odds ratio is not equal to 1
## 95 percent confidence interval:
##  0.2234702       Inf
## sample estimates:
## odds ratio
##        Inf
```

```r
gr <- split(Data2$`Pleural effusion`, Data2$Groups)
results <- lapply(gr, Table.Frequencies)
data.frame(results)
```

```
##   M.Bovis.Niveles M.Bovis.Freq M.Bovis.Freq_Rela M.Bovis.Freq_Acum
## 1             yes            2         0.1666667                 2
## 2              no           10         0.8333333                12
##   M.Bovis.Freq_Rela_Acum M.TB.Niveles M.TB.Freq M.TB.Freq_Rela M.TB.Freq_Acum
## 1              0.1666667          yes         0              0              0
## 2              1.0000000           no        14              1             14
##   M.TB.Freq_Rela_Acum
## 1                   0
## 2                   1
```

```r
table(Data2$Bronchiectasis, Data2$Groups)
```

```
##
##       M.Bovis M.TB
##   yes       1    1
##   no       11   13
```

```r
x<-table(Data2$Bronchiectasis, Data2$Groups)
fisher.test(x)
```

```
##
##  Fisher's Exact Test for Count Data
##
## data:  x
## p-value = 1
## alternative hypothesis: true odds ratio is not equal to 1
## 95 percent confidence interval:
##   0.01382209 99.72841797
## sample estimates:
## odds ratio
##   1.174215
```

```r
gr <- split(Data2$Bronchiectasis, Data2$Groups)
results <- lapply(gr, Table.Frequencies)
data.frame(results)
```

```
##   M.Bovis.Niveles M.Bovis.Freq M.Bovis.Freq_Rela M.Bovis.Freq_Acum
## 1             yes            1        0.08333333                 1
## 2              no           11        0.91666667                12
##   M.Bovis.Freq_Rela_Acum M.TB.Niveles M.TB.Freq M.TB.Freq_Rela M.TB.Freq_Acum
## 1             0.08333333          yes         1     0.07142857              1
## 2             1.00000000           no        13     0.92857143             14
##   M.TB.Freq_Rela_Acum
## 1          0.07142857
## 2          1.00000000
```

```r
table(Data2$`Retroperitoneal lymphadenopathy`, Data2$Groups)
```

```
##
##       M.Bovis M.TB
##   yes      10    3
##   no        2   11
```

```r
x<-table(Data2$`Retroperitoneal lymphadenopathy`, Data2$Groups)
fisher.test(x)
```

```
##
##  Fisher's Exact Test for Count Data
##
## data:  x
## p-value = 0.004832
## alternative hypothesis: true odds ratio is not equal to 1
## 95 percent confidence interval:
##    1.947572 227.519260
## sample estimates:
## odds ratio
##   15.72024
```

```r
gr <- split(Data2$`Retroperitoneal lymphadenopathy`, Data2$Groups)
results <- lapply(gr, Table.Frequencies)
data.frame(results)
```

```
##   M.Bovis.Niveles M.Bovis.Freq M.Bovis.Freq_Rela M.Bovis.Freq_Acum
## 1             yes           10         0.8333333                10
## 2              no            2         0.1666667                12
##   M.Bovis.Freq_Rela_Acum M.TB.Niveles M.TB.Freq M.TB.Freq_Rela M.TB.Freq_Acum
## 1              0.8333333          yes         3      0.2142857              3
## 2              1.0000000           no        11      0.7857143             14
##   M.TB.Freq_Rela_Acum
## 1           0.2142857
## 2           1.0000000
```

```r
table(Data2$`Collections in psoas`, Data2$Groups)
```

```
##
##       M.Bovis M.TB
##   yes       4    2
##   no        8   12
```

```r
x<-table(Data2$`Collections in psoas`, Data2$Groups)
fisher.test(x)
```

```
##
##  Fisher's Exact Test for Count Data
##
## data:  x
```

```
## p-value = 0.3652
## alternative hypothesis: true odds ratio is not equal to 1
## 95 percent confidence interval:
##   0.3210422 39.0160802
## sample estimates:
## odds ratio
##   2.872726
```

```
gr <- split(Data2$`Collections in psoas`, Data2$Groups)
results <- lapply(gr, Table.Frequencies)
data.frame(results)
```

```
##   M.Bovis.Niveles M.Bovis.Freq M.Bovis.Freq_Rela M.Bovis.Freq_Acum
## 1             yes            4         0.3333333                 4
## 2              no            8         0.6666667                12
##   M.Bovis.Freq_Rela_Acum M.TB.Niveles M.TB.Freq M.TB.Freq_Rela M.TB.Freq_Acum
## 1              0.3333333          yes         2      0.1428571              2
## 2              1.0000000           no        12      0.8571429             14
##   M.TB.Freq_Rela_Acum
## 1           0.1428571
## 2           1.0000000
```

```
table(Data2$Hepatomegaly, Data2$Groups)
```

```
##
##       M.Bovis M.TB
##   yes       7    0
##   no        5   14
```

```
x<-table(Data2$Hepatomegaly, Data2$Groups)
fisher.test(x)
```

```
##
##  Fisher's Exact Test for Count Data
##
## data:  x
## p-value = 0.001204
## alternative hypothesis: true odds ratio is not equal to 1
## 95 percent confidence interval:
##  2.633903      Inf
## sample estimates:
## odds ratio
##        Inf
```

```
gr <- split(Data2$Hepatomegaly, Data2$Groups)
results <- lapply(gr, Table.Frequencies)
data.frame(results)
```

```
##   M.Bovis.Niveles M.Bovis.Freq M.Bovis.Freq_Rela M.Bovis.Freq_Acum
## 1             yes            7         0.5833333                 7
## 2              no            5         0.4166667                12
```

```
##   M.Bovis.Freq_Rela_Acum M.TB.Niveles M.TB.Freq M.TB.Freq_Rela M.TB.Freq_Acum
## 1              0.5833333          yes         0              0              0
## 2              1.0000000           no        14              1             14
##   M.TB.Freq_Rela_Acum
## 1                   0
## 2                   1
```

```
table(Data2$Splenomegaly, Data2$Groups)
```

```
##
##       M.Bovis M.TB
##   yes       8    0
##   no        4   14
```

```
x<-table(Data2$Splenomegaly, Data2$Groups)
fisher.test(x)
```

```
##
##  Fisher's Exact Test for Count Data
##
## data:  x
## p-value = 0.0003168
## alternative hypothesis: true odds ratio is not equal to 1
## 95 percent confidence interval:
##  3.615341      Inf
## sample estimates:
## odds ratio
##        Inf
```

```
gr <- split(Data2$Splenomegaly, Data2$Groups)
results <- lapply(gr, Table.Frequencies)
data.frame(results)
```

```
##   M.Bovis.Niveles M.Bovis.Freq M.Bovis.Freq_Rela M.Bovis.Freq_Acum
## 1             yes            8         0.6666667                 8
## 2              no            4         0.3333333                12
##   M.Bovis.Freq_Rela_Acum M.TB.Niveles M.TB.Freq M.TB.Freq_Rela M.TB.Freq_Acum
## 1              0.6666667          yes         0              0              0
## 2              1.0000000           no        14              1             14
##   M.TB.Freq_Rela_Acum
## 1                   0
## 2                   1
```

```
table(Data2$`Splenic abscesses`, Data2$Groups)
```

```
##
##       M.Bovis M.TB
##   yes       6    0
##   no        6   14
```

```r
x<-table(Data2$`Splenic abscesses`, Data2$Groups)
fisher.test(x)
```

```
##
##  Fisher's Exact Test for Count Data
##
## data:  x
## p-value = 0.004013
## alternative hypothesis: true odds ratio is not equal to 1
## 95 percent confidence interval:
##  1.901178      Inf
## sample estimates:
## odds ratio
##        Inf
```

```r
gr <- split(Data2$`Splenic abscesses`, Data2$Groups)
results <- lapply(gr, Table.Frequencies)
data.frame(results)
```

```
##   M.Bovis.Niveles M.Bovis.Freq M.Bovis.Freq_Rela M.Bovis.Freq_Acum
## 1             yes            6               0.5                 6
## 2              no            6               0.5                12
##   M.Bovis.Freq_Rela_Acum M.TB.Niveles M.TB.Freq M.TB.Freq_Rela M.TB.Freq_Acum
## 1                    0.5          yes         0              0              0
## 2                    1.0           no        14              1             14
##   M.TB.Freq_Rela_Acum
## 1                   0
## 2                   1
```

```r
table(Data2$`IS Pulmonary`, Data2$Groups)
```

```
##
##       M.Bovis M.TB
##   yes       2    7
##   no       10    7
```

```r
x<-table(Data2$`IS Pulmonary`, Data2$Groups)
fisher.test(x)
```

```
##
##  Fisher's Exact Test for Count Data
##
## data:  x
## p-value = 0.11
## alternative hypothesis: true odds ratio is not equal to 1
## 95 percent confidence interval:
##  0.01675295 1.59035253
## sample estimates:
## odds ratio
##  0.2132506
```

```r
gr <- split(Data2$`IS Pulmonary`, Data2$Groups)
results <- lapply(gr, Table.Frequencies)
data.frame(results)
```

```
##   M.Bovis.Niveles M.Bovis.Freq M.Bovis.Freq_Rela M.Bovis.Freq_Acum
## 1             yes            2         0.1666667                 2
## 2              no           10         0.8333333                12
##   M.Bovis.Freq_Rela_Acum M.TB.Niveles M.TB.Freq M.TB.Freq_Rela M.TB.Freq_Acum
## 1              0.1666667          yes         7            0.5              7
## 2              1.0000000           no         7            0.5             14
##   M.TB.Freq_Rela_Acum
## 1                 0.5
## 2                 1.0
```

```r
table(Data2$`IS Ganglionar`, Data2$Groups)
```

```
##
##       M.Bovis M.TB
##   yes       5    4
##   no        7   10
```

```r
x<-table(Data2$`IS Ganglionar`, Data2$Groups)
fisher.test(x)
```

```
##
##  Fisher's Exact Test for Count Data
##
## data:  x
## p-value = 0.6828
## alternative hypothesis: true odds ratio is not equal to 1
## 95 percent confidence interval:
##   0.2638369 12.4750190
## sample estimates:
## odds ratio
##   1.745706
```

```r
gr <- split(Data2$`IS Ganglionar`, Data2$Groups)
results <- lapply(gr, Table.Frequencies)
data.frame(results)
```

```
##   M.Bovis.Niveles M.Bovis.Freq M.Bovis.Freq_Rela M.Bovis.Freq_Acum
## 1             yes            5         0.4166667                 5
## 2              no            7         0.5833333                12
##   M.Bovis.Freq_Rela_Acum M.TB.Niveles M.TB.Freq M.TB.Freq_Rela M.TB.Freq_Acum
## 1              0.4166667          yes         4      0.2857143              4
## 2              1.0000000           no        10      0.7142857             14
##   M.TB.Freq_Rela_Acum
## 1           0.2857143
## 2           1.0000000
```

```r
table(Data2$`IS Abdominal`, Data2$Groups)
```

```
##
##       M.Bovis M.TB
##   yes       3    3
##   no        9   11
```

```r
x<-table(Data2$`IS Abdominal`, Data2$Groups)
fisher.test(x)
```

```
##
##  Fisher's Exact Test for Count Data
##
## data:  x
## p-value = 1
## alternative hypothesis: true odds ratio is not equal to 1
## 95 percent confidence interval:
##   0.1289578 11.4470359
## sample estimates:
## odds ratio
##   1.212778
```

```r
gr <- split(Data2$`IS Abdominal`, Data2$Groups)
results <- lapply(gr, Table.Frequencies)
data.frame(results)
```

```
##   M.Bovis.Niveles M.Bovis.Freq M.Bovis.Freq_Rela M.Bovis.Freq_Acum
## 1             yes            3              0.25                 3
## 2              no            9              0.75                12
##   M.Bovis.Freq_Rela_Acum M.TB.Niveles M.TB.Freq M.TB.Freq_Rela M.TB.Freq_Acum
## 1                   0.25          yes         3      0.2142857              3
## 2                   1.00           no        11      0.7857143             14
##   M.TB.Freq_Rela_Acum
## 1           0.2142857
## 2           1.0000000
```

```r
table(Data2$`IS CSF`, Data2$Groups)
```

```
##
##       M.Bovis M.TB
##   yes       0    2
##   no       12   12
```

```r
x<-table(Data2$`IS CSF`, Data2$Groups)
fisher.test(x)
```

```
##
##  Fisher's Exact Test for Count Data
##
## data:  x
```

```
## p-value = 0.4831
## alternative hypothesis: true odds ratio is not equal to 1
## 95 percent confidence interval:
##  0.000000 6.169903
## sample estimates:
## odds ratio
##          0
```

```
gr <- split(Data2$`IS CSF`, Data2$Groups)
results <- lapply(gr, Table.Frequencies)
data.frame(results)
```

```
##   M.Bovis.Niveles M.Bovis.Freq M.Bovis.Freq_Rela M.Bovis.Freq_Acum
## 1             yes            0                 0                 0
## 2              no           12                 1                12
##   M.Bovis.Freq_Rela_Acum M.TB.Niveles M.TB.Freq M.TB.Freq_Rela M.TB.Freq_Acum
## 1                      0          yes         2      0.1428571              2
## 2                      1           no        12      0.8571429             14
##   M.TB.Freq_Rela_Acum
## 1           0.1428571
## 2           1.0000000
```

```
table(Data2$`IS Psoas`, Data2$Groups)
```

```
##
##       M.Bovis M.TB
##   yes       1    1
##   no       11   13
```

```
x<-table(Data2$`IS Psoas`, Data2$Groups)
fisher.test(x)
```

```
##
##  Fisher's Exact Test for Count Data
##
## data:  x
## p-value = 1
## alternative hypothesis: true odds ratio is not equal to 1
## 95 percent confidence interval:
##   0.01382209 99.72841797
## sample estimates:
## odds ratio
##   1.174215
```

```
gr <- split(Data2$`IS Psoas`, Data2$Groups)
results <- lapply(gr, Table.Frequencies)
data.frame(results)
```

```
##   M.Bovis.Niveles M.Bovis.Freq M.Bovis.Freq_Rela M.Bovis.Freq_Acum
## 1             yes            1        0.08333333                 1
## 2              no           11        0.91666667                12
```

```
##   M.Bovis.Freq_Rela_Acum M.TB.Niveles M.TB.Freq M.TB.Freq_Rela M.TB.Freq_Acum
## 1             0.08333333          yes         1     0.07142857              1
## 2             1.00000000           no        13     0.92857143             14
##   M.TB.Freq_Rela_Acum
## 1          0.07142857
## 2          1.00000000
```

```
table(Data2$`IS Genitourinary`, Data2$Groups)
```

```
##
##       M.Bovis M.TB
##   yes       0    0
##   no       12   14
```

```
x<-table(Data2$`IS Genitourinary`, Data2$Groups)
fisher.test(x)
```

```
##
##  Fisher's Exact Test for Count Data
##
## data:  x
## p-value = 1
## alternative hypothesis: true odds ratio is not equal to 1
## 95 percent confidence interval:
##    0 Inf
## sample estimates:
## odds ratio
##          0
```

```
gr <- split(Data2$`IS Genitourinary`, Data2$Groups)
results <- lapply(gr, Table.Frequencies)
data.frame(results)
```

```
##   M.Bovis.Niveles M.Bovis.Freq M.Bovis.Freq_Rela M.Bovis.Freq_Acum
## 1             yes            0                 0                 0
## 2              no           12                 1                12
##   M.Bovis.Freq_Rela_Acum M.TB.Niveles M.TB.Freq M.TB.Freq_Rela M.TB.Freq_Acum
## 1                      0          yes         0              0              0
## 2                      1           no        14              1             14
##   M.TB.Freq_Rela_Acum
## 1                   0
## 2                   1
```

```
table(Data2$`IS Bones`, Data2$Groups)
```

```
##
##       M.Bovis M.TB
##   yes       2    1
##   no       10   13
```

```r
x<-table(Data2$`IS Bones`, Data2$Groups)
fisher.test(x)
```

```
##
##  Fisher's Exact Test for Count Data
##
## data:  x
## p-value = 0.58
## alternative hypothesis: true odds ratio is not equal to 1
## 95 percent confidence interval:
##    0.1149909 164.7865129
## sample estimates:
## odds ratio
##   2.506744
```

```r
gr <- split(Data2$`IS Bones`, Data2$Groups)
results <- lapply(gr, Table.Frequencies)
data.frame(results)
```

```
##   M.Bovis.Niveles M.Bovis.Freq M.Bovis.Freq_Rela M.Bovis.Freq_Acum
## 1             yes            2         0.1666667                 2
## 2              no           10         0.8333333                12
##   M.Bovis.Freq_Rela_Acum M.TB.Niveles M.TB.Freq M.TB.Freq_Rela M.TB.Freq_Acum
## 1              0.1666667          yes         1     0.07142857              1
## 2              1.0000000           no        13     0.92857143             14
##   M.TB.Freq_Rela_Acum
## 1          0.07142857
## 2          1.00000000
```

```r
table(Data2$`Xpert MTb/RIF`, Data2$Groups)
```

```
##
##       M.Bovis M.TB
##   yes      11   12
##   no        1    2
```

```r
x<-table(Data2$`Xpert MTb/RIF`, Data2$Groups)
fisher.test(x)
```

```
##
##  Fisher's Exact Test for Count Data
##
## data:  x
## p-value = 1
## alternative hypothesis: true odds ratio is not equal to 1
## 95 percent confidence interval:
##    0.08224634 117.74859949
## sample estimates:
## odds ratio
##    1.79243
```

```
gr <- split(Data2$`Xpert MTb/RIF`, Data2$Groups)
results <- lapply(gr, Table.Frequencies)
data.frame(results)
```

```
##   M.Bovis.Niveles M.Bovis.Freq M.Bovis.Freq_Rela M.Bovis.Freq_Acum
## 1             yes           11        0.91666667                11
## 2              no            1        0.08333333                12
##   M.Bovis.Freq_Rela_Acum M.TB.Niveles M.TB.Freq M.TB.Freq_Rela M.TB.Freq_Acum
## 1              0.9166667          yes        12      0.8571429             12
## 2              1.0000000           no         2      0.1428571             14
##   M.TB.Freq_Rela_Acum
## 1           0.8571429
## 2           1.0000000
```

```
table(Data2$`Resistance to rifampicin by Xpert MTb/RIF`, Data2$Groups)
```

```
##
##       M.Bovis M.TB
##   yes       0    0
##   no       12   14
```

```
x<-table(Data2$Cough, Data2$Groups)
fisher.test(x)
```

```
##
##  Fisher's Exact Test for Count Data
##
## data:  x
## p-value = 0.2671
## alternative hypothesis: true odds ratio is not equal to 1
## 95 percent confidence interval:
##   0.4223446 17.9834426
## sample estimates:
## odds ratio
##   2.564529
```

```
gr <- split(Data2$`Resistance to rifampicin by Xpert MTb/RIF`, Data2$Groups)
results <- lapply(gr, Table.Frequencies)
data.frame(results)
```

```
##   M.Bovis.Niveles M.Bovis.Freq M.Bovis.Freq_Rela M.Bovis.Freq_Acum
## 1             yes            0                 0                 0
## 2              no           12                 1                12
##   M.Bovis.Freq_Rela_Acum M.TB.Niveles M.TB.Freq M.TB.Freq_Rela M.TB.Freq_Acum
## 1                      0          yes         0              0              0
## 2                      1           no        14              1             14
##   M.TB.Freq_Rela_Acum
## 1                   0
## 2                   1
```

```
table(Data2$Tinction, Data2$Groups)
```

```
##
##       M.Bovis M.TB
##   yes      10    5
##   no        2    9
```

```
x<-table(Data2$Tinction, Data2$Groups)
fisher.test(x)
```

```
##
##  Fisher's Exact Test for Count Data
##
## data:  x
## p-value = 0.02142
## alternative hypothesis: true odds ratio is not equal to 1
## 95 percent confidence interval:
##    1.098346 106.449047
## sample estimates:
## odds ratio
##   8.167933
```

```
gr <- split(Data2$Tinction, Data2$Groups)
results <- lapply(gr, Table.Frequencies)
data.frame(results)
```

```
##   M.Bovis.Niveles M.Bovis.Freq M.Bovis.Freq_Rela M.Bovis.Freq_Acum
## 1             yes           10         0.8333333                10
## 2              no            2         0.1666667                12
##   M.Bovis.Freq_Rela_Acum M.TB.Niveles M.TB.Freq M.TB.Freq_Rela M.TB.Freq_Acum
## 1              0.8333333          yes         5      0.3571429              5
## 2              1.0000000           no         9      0.6428571             14
##   M.TB.Freq_Rela_Acum
## 1           0.3571429
## 2           1.0000000
```

```
table(Data2$Histopathology, Data2$Groups)
```

```
##
##       M.Bovis M.TB
##   yes       4    5
##   no        8    9
```

```
x<-table(Data2$Histopathology, Data2$Groups)
fisher.test(x)
```

```
##
##  Fisher's Exact Test for Count Data
##
## data:  x
```

```
## p-value = 1
## alternative hypothesis: true odds ratio is not equal to 1
## 95 percent confidence interval:
##  0.1288941 6.0041037
## sample estimates:
## odds ratio
##  0.9036515
```

```
gr <- split(Data2$Histopathology, Data2$Groups)
results <- lapply(gr, Table.Frequencies)
data.frame(results)
```

```
##   M.Bovis.Niveles M.Bovis.Freq M.Bovis.Freq_Rela M.Bovis.Freq_Acum
## 1             yes            4         0.3333333                 4
## 2              no            8         0.6666667                12
##   M.Bovis.Freq_Rela_Acum M.TB.Niveles M.TB.Freq M.TB.Freq_Rela M.TB.Freq_Acum
## 1              0.3333333          yes         5      0.3571429              5
## 2              1.0000000           no         9      0.6428571             14
##   M.TB.Freq_Rela_Acum
## 1           0.3571429
## 2           1.0000000
```

```
normality<-function(x){
  car::qqPlot(x)
  boxplot(x)
  a<-shapiro.test(x)
  b<-FSA::ksTest(x,"pnorm", mean(x), sd(x))
  print(a)
  print(b)
  }
```

```
normality(Data2$`Crops grown`)
```

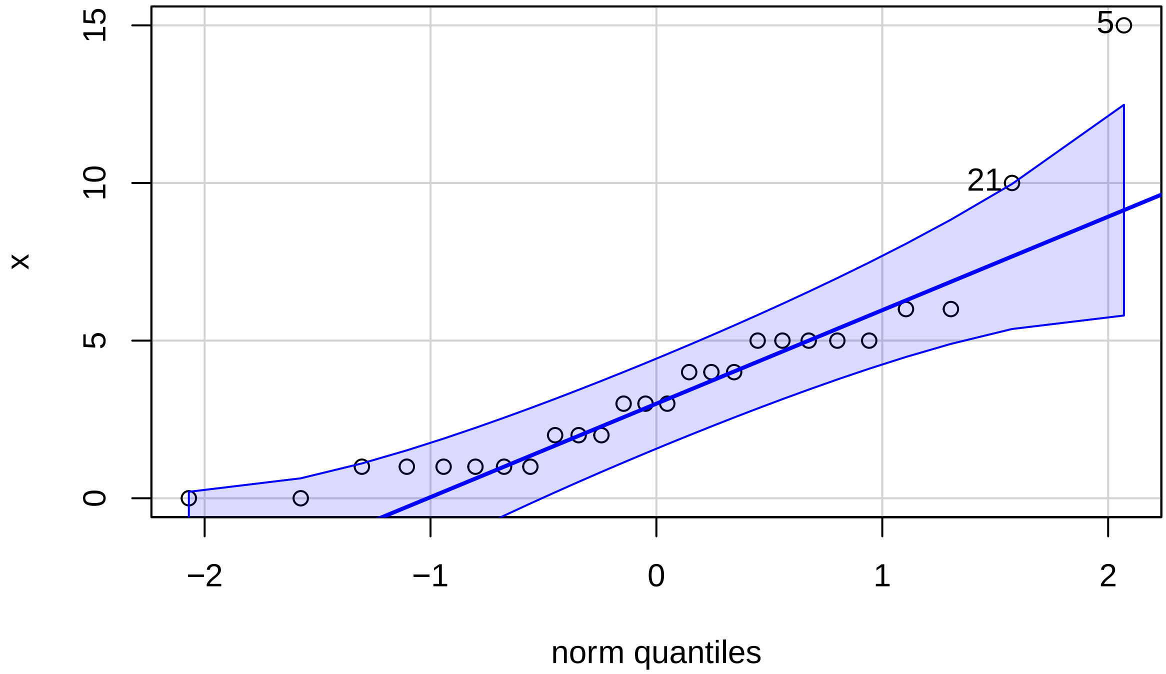


```
## Registered S3 methods overwritten by 'FSA':
##   method       from
##   confint.boot car
##   hist.boot    car
```

```
## Warning in stats::ks.test(x, y, ..., alternative = alternative, exact = exact):
## ties should not be present for the Kolmogorov-Smirnov test
```

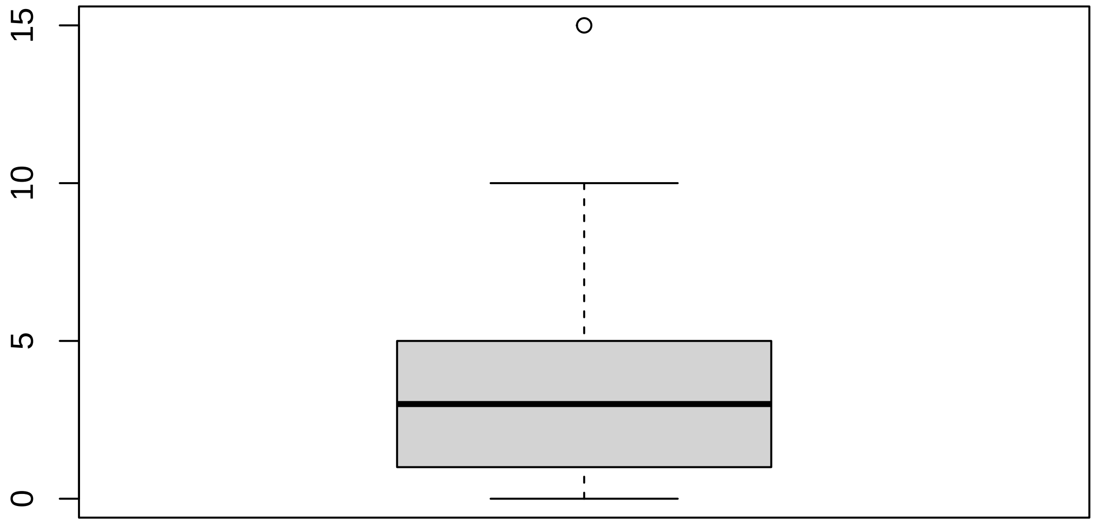


```
##
##  Shapiro-Wilk normality test
##
## data:  x
## W = 0.82118, p-value = 0.0004127
##
##
##  One-sample Kolmogorov-Smirnov test
##
## data:  x
## D = 0.1866, p-value = 0.3257
## alternative hypothesis: two-sided
```

```
normality(Data2$`Positive growns`)
```

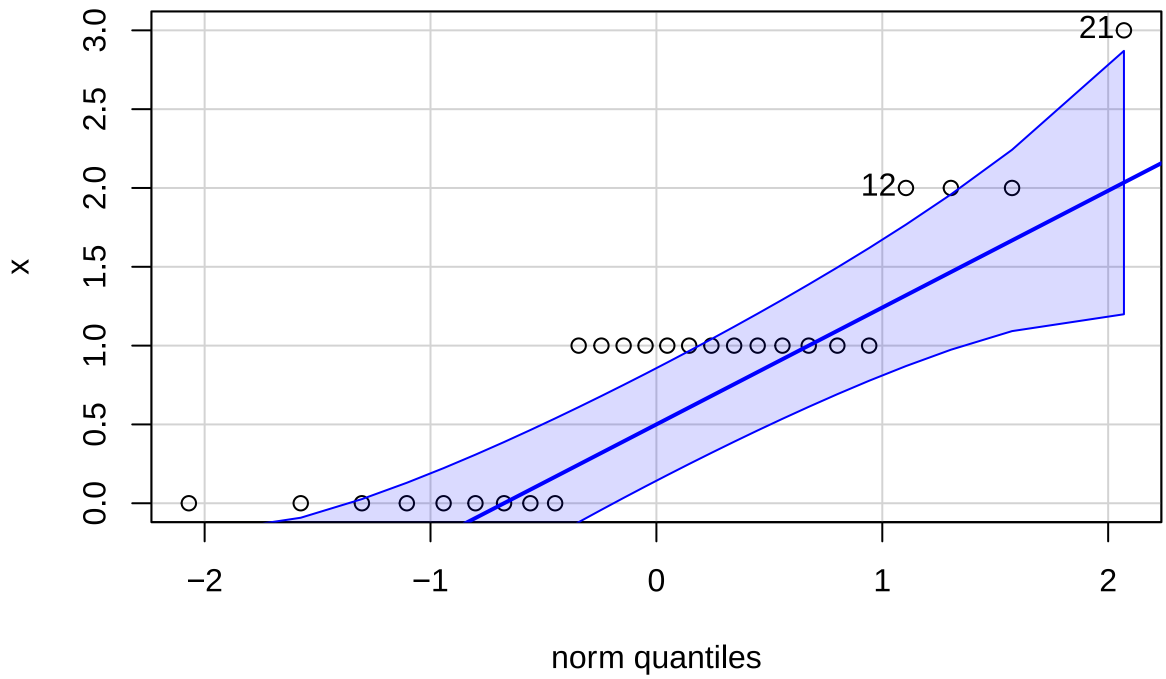


```
## Warning in stats::ks.test(x, y, ..., alternative = alternative, exact = exact):
## ties should not be present for the Kolmogorov-Smirnov test
```

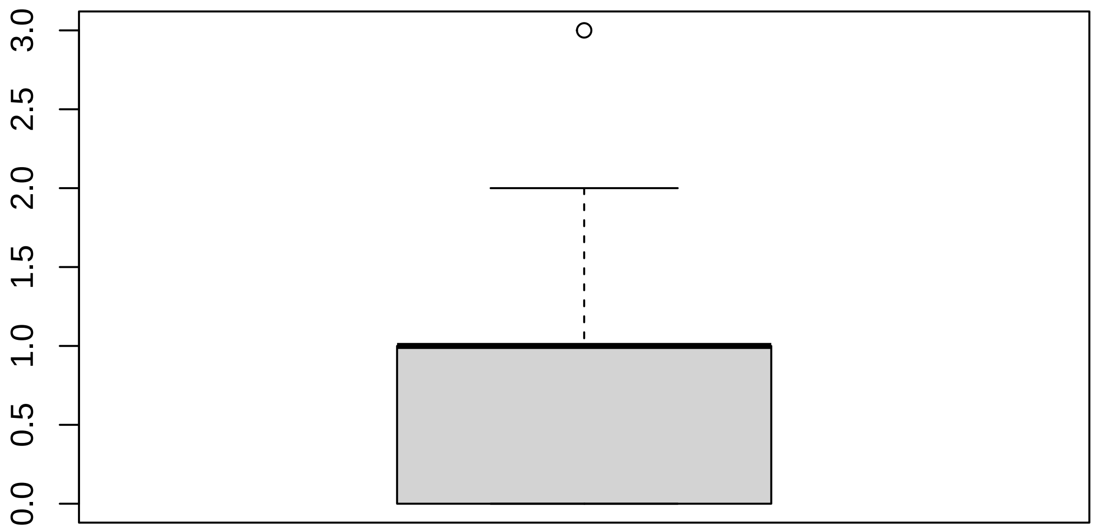


```
##
##  Shapiro-Wilk normality test
##
## data:  x
## W = 0.81259, p-value = 0.0002905
##
##
##  One-sample Kolmogorov-Smirnov test
##
## data:  x
## D = 0.26841, p-value = 0.04721
## alternative hypothesis: two-sided
```

```
no.norm.analysis <- function(x, y){
  a <- FSA::Summarize(x ~ y)
  b <- wilcox.test(x ~ y, paired = F)
  cat("\nDescriptivos:\n")
  print(a)
  cat("\nU Mann Whitney:\n")
  print(b)
  boxplot(x~y)
}
```

```
no.norm.analysis(Data2$`Crops grown`, Data2$Groups)
```

```
## Warning in wilcox.test.default(x = c(1, 5, 6, 0, 15, 4, 5, 1, 0, 1, 1, 6: cannot
## compute exact p-value with ties
```

```
##
## Descriptivos:
##          y  n     mean       sd min Q1 median   Q3 max percZero
## 1 M.Bovis 12 3.750000 4.245318   0  1    2.5 5.25  15 16.66667
## 2    M.TB 14 3.571429 2.310987   1  2    3.0 4.75  10  0.00000
##
## U Mann Whitney:
##
##  Wilcoxon rank sum test with continuity correction
##
## data:  x by y
## W = 76, p-value = 0.6962
## alternative hypothesis: true location shift is not equal to 0
```

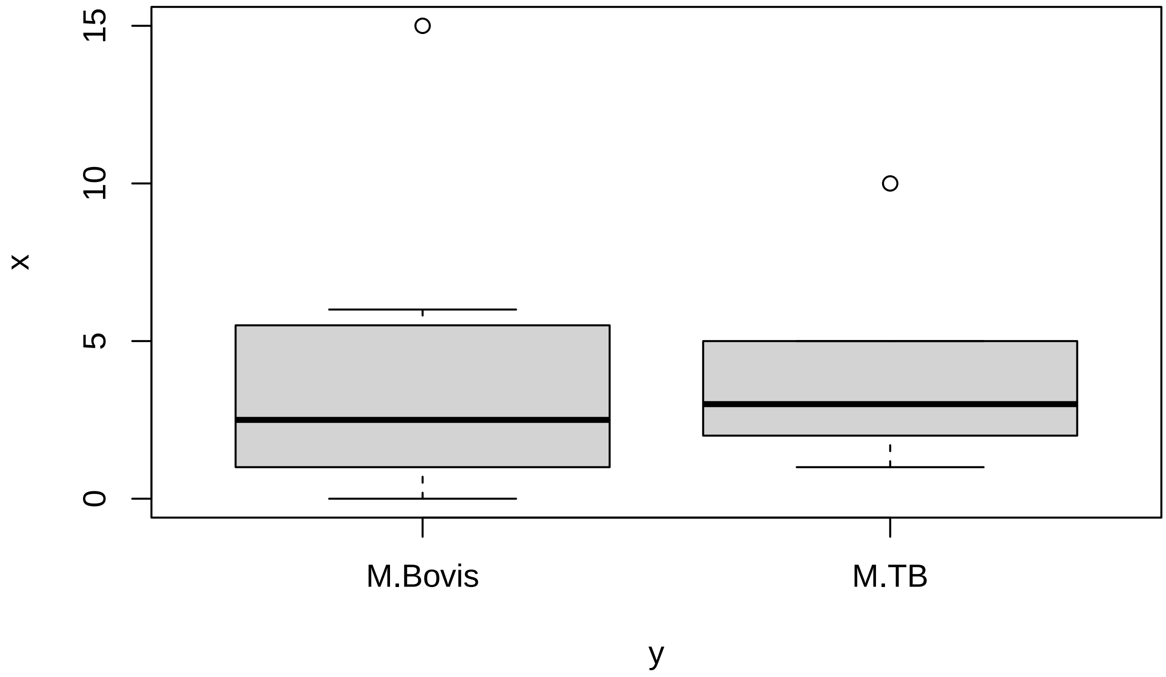


```
no.norm.analysis(Data2$`Positive growns`, Data2$Groups)
```

```
## Warning in wilcox.test.default(x = c(1, 0, 0, 0, 0, 0, 0, 0, 0, 1, 0, 2), :
## cannot compute exact p-value with ties
```

```
##
## Descriptivos:
```

```
##          y  n      mean        sd min Q1 median   Q3 max percZero
## 1 M.Bovis 12 0.3333333 0.6513389   0  0      0 0.25   2       75
## 2    M.TB 14 1.2857143 0.6112498   1  1      1 1.00   3        0
##
## U Mann Whitney:
##
##  Wilcoxon rank sum test with continuity correction
##
## data:  x by y
## W = 23, p-value = 0.0006517
## alternative hypothesis: true location shift is not equal to 0
```

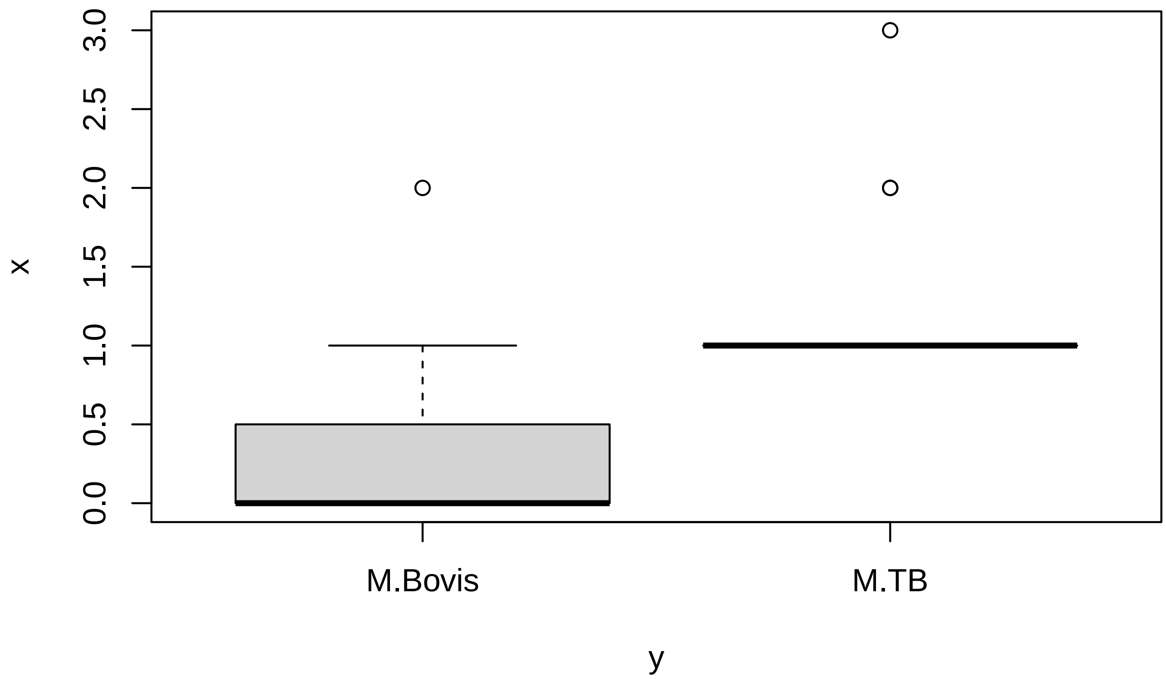

Treatment and outcome of patients with M tuberculosis or M. bovis infection in HIV-infected subjects seen at HCGFAA.

Andrea Torres Rojas

2025-06-03

```
library(readxl)
Data3 <- read_excel("C:/Users/andre/OneDrive/Escritorio/Data3.xlsx")
head(Data3)
```

```
## # A tibble: 6 x 18
##   Groups    ID ‘Cure at first treatment‘ ‘failed first t~ relapse ‘re treatment‘
##    <dbl> <dbl>                     <dbl>            <dbl>   <dbl>          <dbl>
## 1      1     1                         2                2       2              1
## 2      1     2                         2                2       1              1
## 3      1     3                         2                2       1              1
## 4      1     4                         2                1       2              1
## 5      1     5                         2                2       2              1
## 6      1     6                         2                2       2              1
## # ... with 12 more variables: cure at 2nd treatment <dbl>,
## #   number of treatment <dbl>, Adherence <dbl>, qx drained <dbl>,
## #   hospitalization <dbl>, days in hospital <dbl>, cured outcome <dbl>,
## #   on treatment <dbl>, failure <dbl>, outcome relapse <dbl>,
## #   lost of follow up <dbl>, death <dbl>
```

```
View(Data3)
```

```
Data3$Groups<-as.factor(Data3$Groups)
Data3$Groups<-factor(Data3$Groups,levels = c(1,2), labels = c("M.Bovis", "M.TB"))
Data3$`failed first treatment`<-as.factor(Data3$`failed first treatment`)
Data3$`failed first treatment`<-factor(Data3$`failed first treatment`, levels = c(1,2), labels = c("yes
Data3$`Cure at first treatment`<-as.factor(Data3$`Cure at first treatment`)
Data3$`Cure at first treatment`<-factor(Data3$`Cure at first treatment`, levels = c(1,2), labels = c("y
Data3$relapse<-as.factor(Data3$relapse)
Data3$relapse<-factor(Data3$relapse, levels = c(1,2), labels = c("yes", "no"))
Data3$`re treatment`<-as.factor(Data3$`re treatment`)
Data3$`re treatment`<-factor(Data3$`re treatment`, levels = c(1,2), labels = c("yes", "no"))
Data3$`cure at 2nd treatment`<-as.factor(Data3$`cure at 2nd treatment`)
Data3$`cure at 2nd treatment`<-factor(Data3$`cure at 2nd treatment`, levels = c(1,2), labels = c("yes",
Data3$Adherence<-as.factor(Data3$Adherence)
Data3$Adherence<-factor(Data3$Adherence, levels = c(1,2), labels = c("yes", "no"))
Data3$`qx drained`<-as.factor(Data3$`qx drained`)
Data3$`qx drained`<-factor(Data3$`qx drained`, levels = c(1,2), labels = c("yes", "no"))
Data3$`cured outcome`<-as.factor(Data3$`cured outcome`)
Data3$`cured outcome`<-factor(Data3$`cured outcome`, levels = c(1,2), labels = c("yes", "no"))
```

```r
Data3$`on treatment`<-as.factor(Data3$`on treatment`)
Data3$`on treatment`<-factor(Data3$`on treatment`, levels = c(1,2), labels = c("yes", "no"))
Data3$failure<-as.factor(Data3$failure)
Data3$failure<-factor(Data3$failure, levels = c(1,2), labels = c("yes", "no"))
Data3$`outcome relapse`<-as.factor(Data3$`outcome relapse`)
Data3$`outcome relapse`<-factor(Data3$`outcome relapse`, levels = c(1,2), labels = c("yes", "no"))
Data3$`lost of follow up`<-as.factor(Data3$`lost of follow up`)
Data3$`lost of follow up`<-factor(Data3$`lost of follow up`, levels = c(1,2), labels = c("yes", "no"))
Data3$death<-as.factor(Data3$death)
Data3$death<-factor(Data3$death, levels = c(1,2), labels = c("yes", "no"))
```

```r
str(Data3)
```

```
## tibble [26 x 18] (S3: tbl_df/tbl/data.frame)
##  $ Groups                 : Factor w/ 2 levels "M.Bovis","M.TB": 1 1 1 1 1 1 1 1 1 1 ...
##  $ ID                     : num [1:26] 1 2 3 4 5 6 7 8 9 10 ...
##  $ Cure at first treatment: Factor w/ 2 levels "yes","no": 2 2 2 2 2 2 2 2 2 2 ...
##  $ failed first treatment : Factor w/ 2 levels "yes","no": 2 2 2 1 2 2 2 2 2 2 ...
##  $ relapse                : Factor w/ 2 levels "yes","no": 2 1 1 2 2 2 2 2 2 2 ...
##  $ re treatment           : Factor w/ 2 levels "yes","no": 1 1 1 1 1 1 1 1 1 1 ...
##  $ cure at 2nd treatment  : Factor w/ 2 levels "yes","no": 2 2 2 2 2 2 2 2 2 2 ...
##  $ number of treatment    : num [1:26] 2 4 2 2 3 2 2 2 1 2 ...
##  $ Adherence              : Factor w/ 2 levels "yes","no": 2 2 1 1 2 1 1 1 1 2 ...
##  $ qx drained             : Factor w/ 2 levels "yes","no": 1 2 1 1 1 1 1 2 1 2 ...
##  $ hospitalization        : num [1:26] 0 4 1 1 3 2 1 2 1 3 ...
##  $ days in hospital       : num [1:26] 0 31 19 29 24 7 4 11 6 40 ...
##  $ cured outcome          : Factor w/ 2 levels "yes","no": 2 2 1 1 2 1 1 1 1 2 ...
##  $ on treatment           : Factor w/ 2 levels "yes","no": 2 2 2 2 2 2 2 2 2 2 ...
##  $ failure                : Factor w/ 2 levels "yes","no": 1 1 2 2 2 2 2 2 2 2 ...
##  $ outcome relapse        : Factor w/ 2 levels "yes","no": 2 2 2 2 2 2 2 2 2 2 ...
##  $ lost of follow up      : Factor w/ 2 levels "yes","no": 2 2 2 2 1 2 2 2 2 2 ...
##  $ death                  : Factor w/ 2 levels "yes","no": 2 2 2 2 2 2 2 2 2 1 ...
```

```r
Table.Frequencies <- function(x){
  y <- table(x)
  data_freq <-
    data.frame(Niveles= names(y),
               Freq =as.numeric(y),
               Freq_Rela = as.numeric(prop.table(y)),
               Freq_Acum = as.numeric(cumsum(y)),
               Freq_Rela_Acum = as.numeric (cumsum(prop.table(y))))

  data_freq}
```

```r
table(Data3$`Cure at first treatment`, Data3$Groups)
```

```
##
##       M.Bovis M.TB
##   yes       0    3
##   no       12   11
```

```r
x<-table(Data3$`Cure at first treatment`, Data3$Groups)
fisher.test(x)
```

```
##
##  Fisher's Exact Test for Count Data
##
## data:  x
## p-value = 0.2246
## alternative hypothesis: true odds ratio is not equal to 1
## 95 percent confidence interval:
##  0.000000 2.716601
## sample estimates:
## odds ratio
##          0
```

```r
gr <- split(Data3$`Cure at first treatment`, Data3$Groups)
results <- lapply(gr, Table.Frequencies)
data.frame(results)
```

```
##   M.Bovis.Niveles M.Bovis.Freq M.Bovis.Freq_Rela M.Bovis.Freq_Acum
## 1             yes            0                 0                 0
## 2              no           12                 1                12
##   M.Bovis.Freq_Rela_Acum M.TB.Niveles M.TB.Freq M.TB.Freq_Rela M.TB.Freq_Acum
## 1                      0          yes         3      0.2142857              3
## 2                      1           no        11      0.7857143             14
##   M.TB.Freq_Rela_Acum
## 1           0.2142857
## 2           1.0000000
```

```r
table(Data3$`failed first treatment`, Data3$Groups)
```

```
##
##       M.Bovis M.TB
##   yes       2    2
##   no       10   12
```

```r
x<-table(Data3$`failed first treatment`, Data3$Groups)
fisher.test(x)
```

```
##
##  Fisher's Exact Test for Count Data
##
## data:  x
## p-value = 1
## alternative hypothesis: true odds ratio is not equal to 1
## 95 percent confidence interval:
##   0.07375768 19.27169094
## sample estimates:
## odds ratio
##   1.191575
```

```r
gr <- split(Data3$`failed first treatment`, Data3$Groups)
results <- lapply(gr, Table.Frequencies)
data.frame(results)
```

```
##   M.Bovis.Niveles M.Bovis.Freq M.Bovis.Freq_Rela M.Bovis.Freq_Acum
## 1             yes            2         0.1666667                 2
## 2              no           10         0.8333333                12
##   M.Bovis.Freq_Rela_Acum M.TB.Niveles M.TB.Freq M.TB.Freq_Rela M.TB.Freq_Acum
## 1              0.1666667          yes         2      0.1428571              2
## 2              1.0000000           no        12      0.8571429             14
##   M.TB.Freq_Rela_Acum
## 1           0.1428571
## 2           1.0000000
```

```r
table(Data3$relapse, Data3$Groups)
```

```
##
##       M.Bovis M.TB
##   yes       2    1
##   no       10   13
```

```r
x<-table(Data3$relapse, Data3$Groups)
fisher.test(x)
```

```
##
##  Fisher's Exact Test for Count Data
##
## data:  x
## p-value = 0.58
## alternative hypothesis: true odds ratio is not equal to 1
## 95 percent confidence interval:
##    0.1149909 164.7865129
## sample estimates:
## odds ratio
##   2.506744
```

```r
gr <- split(Data3$relapse, Data3$Groups)
results <- lapply(gr, Table.Frequencies)
data.frame(results)
```

```
##   M.Bovis.Niveles M.Bovis.Freq M.Bovis.Freq_Rela M.Bovis.Freq_Acum
## 1             yes            2         0.1666667                 2
## 2              no           10         0.8333333                12
##   M.Bovis.Freq_Rela_Acum M.TB.Niveles M.TB.Freq M.TB.Freq_Rela M.TB.Freq_Acum
## 1              0.1666667          yes         1     0.07142857              1
## 2              1.0000000           no        13     0.92857143             14
##   M.TB.Freq_Rela_Acum
## 1          0.07142857
## 2          1.00000000
```

```r
table(Data3$`re treatment`, Data3$Groups)
```

```
##
##       M.Bovis M.TB
##   yes      12    5
##   no        0    9
```

```r
x<-table(Data3$`re treatment`, Data3$Groups)
fisher.test(x)
```

```
##
##  Fisher's Exact Test for Count Data
##
## data:  x
## p-value = 0.0007111
## alternative hypothesis: true odds ratio is not equal to 1
## 95 percent confidence interval:
##  2.993609      Inf
## sample estimates:
## odds ratio
##        Inf
```

```r
gr <- split(Data3$`re treatment`, Data3$Groups)
results <- lapply(gr, Table.Frequencies)
data.frame(results)
```

```
##   M.Bovis.Niveles M.Bovis.Freq M.Bovis.Freq_Rela M.Bovis.Freq_Acum
## 1             yes           12                 1                12
## 2              no            0                 0                12
##   M.Bovis.Freq_Rela_Acum M.TB.Niveles M.TB.Freq M.TB.Freq_Rela M.TB.Freq_Acum
## 1                      1          yes         5      0.3571429              5
## 2                      1           no         9      0.6428571             14
##   M.TB.Freq_Rela_Acum
## 1           0.3571429
## 2           1.0000000
```

```r
table(Data3$`cure at 2nd treatment`, Data3$Groups)
```

```
##
##       M.Bovis M.TB
##   yes       0    0
##   no       12   14
```

```r
x<-table(Data3$`cure at 2nd treatment`, Data3$Groups)
fisher.test(x)
```

```
##
##  Fisher's Exact Test for Count Data
##
## data:  x
```

```
## p-value = 1
## alternative hypothesis: true odds ratio is not equal to 1
## 95 percent confidence interval:
##    0 Inf
## sample estimates:
## odds ratio
##          0
```

```
gr <- split(Data3$`cure at 2nd treatment`, Data3$Groups)
results <- lapply(gr, Table.Frequencies)
data.frame(results)
```

```
##   M.Bovis.Niveles M.Bovis.Freq M.Bovis.Freq_Rela M.Bovis.Freq_Acum
## 1             yes            0                 0                 0
## 2              no           12                 1                12
##   M.Bovis.Freq_Rela_Acum M.TB.Niveles M.TB.Freq M.TB.Freq_Rela M.TB.Freq_Acum
## 1                      0          yes         0              0              0
## 2                      1           no        14              1             14
##   M.TB.Freq_Rela_Acum
## 1                   0
## 2                   1
```

```
table(Data3$Adherence, Data3$Groups)
```

```
##
##       M.Bovis M.TB
##   yes       8    7
##   no        4    7
```

```
x<-table(Data3$Adherence, Data3$Groups)
fisher.test(x)
```

```
##
##  Fisher's Exact Test for Count Data
##
## data:  x
## p-value = 0.4527
## alternative hypothesis: true odds ratio is not equal to 1
## 95 percent confidence interval:
##   0.3182209 13.4255750
## sample estimates:
## odds ratio
##   1.946708
```

```
gr <- split(Data3$Adherence, Data3$Groups)
results <- lapply(gr, Table.Frequencies)
data.frame(results)
```

```
##   M.Bovis.Niveles M.Bovis.Freq M.Bovis.Freq_Rela M.Bovis.Freq_Acum
## 1             yes            8         0.6666667                 8
## 2              no            4         0.3333333                12
```

```
##   M.Bovis.Freq_Rela_Acum M.TB.Niveles M.TB.Freq M.TB.Freq_Rela M.TB.Freq_Acum
## 1              0.6666667          yes         7            0.5              7
## 2              1.0000000           no         7            0.5             14
##   M.TB.Freq_Rela_Acum
## 1                 0.5
## 2                 1.0
```

```
table(Data3$`qx drained`, Data3$Groups)
```

```
##
##       M.Bovis M.TB
##   yes       8    0
##   no        4   14
```

```
x<-table(Data3$`qx drained`, Data3$Groups)
fisher.test(x)
```

```
##
##  Fisher's Exact Test for Count Data
##
## data:  x
## p-value = 0.0003168
## alternative hypothesis: true odds ratio is not equal to 1
## 95 percent confidence interval:
##  3.615341      Inf
## sample estimates:
## odds ratio
##        Inf
```

```
gr <- split(Data3$`qx drained`, Data3$Groups)
results <- lapply(gr, Table.Frequencies)
data.frame(results)
```

```
##   M.Bovis.Niveles M.Bovis.Freq M.Bovis.Freq_Rela M.Bovis.Freq_Acum
## 1             yes            8         0.6666667                 8
## 2              no            4         0.3333333                12
##   M.Bovis.Freq_Rela_Acum M.TB.Niveles M.TB.Freq M.TB.Freq_Rela M.TB.Freq_Acum
## 1              0.6666667          yes         0              0              0
## 2              1.0000000           no        14              1             14
##   M.TB.Freq_Rela_Acum
## 1                   0
## 2                   1
```

```
table(Data3$`cured outcome`, Data3$Groups)
```

```
##
##       M.Bovis M.TB
##   yes       8    7
##   no        4    7
```

```r
x<-table(Data3$`cured outcome`, Data3$Groups)
fisher.test(x)
```

```
##
##  Fisher's Exact Test for Count Data
##
## data:  x
## p-value = 0.4527
## alternative hypothesis: true odds ratio is not equal to 1
## 95 percent confidence interval:
##   0.3182209 13.4255750
## sample estimates:
## odds ratio
##   1.946708
```

```r
gr <- split(Data3$`cured outcome`, Data3$Groups)
results <- lapply(gr, Table.Frequencies)
data.frame(results)
```

```
##   M.Bovis.Niveles M.Bovis.Freq M.Bovis.Freq_Rela M.Bovis.Freq_Acum
## 1             yes            8         0.6666667                 8
## 2              no            4         0.3333333                12
##   M.Bovis.Freq_Rela_Acum M.TB.Niveles M.TB.Freq M.TB.Freq_Rela M.TB.Freq_Acum
## 1              0.6666667          yes         7            0.5              7
## 2              1.0000000           no         7            0.5             14
##   M.TB.Freq_Rela_Acum
## 1                 0.5
## 2                 1.0
```

```r
table(Data3$`on treatment`, Data3$Groups)
```

```
##
##       M.Bovis M.TB
##   yes       1    0
##   no       11   14
```

```r
x<-table(Data3$`on treatment`, Data3$Groups)
fisher.test(x)
```

```
##
##  Fisher's Exact Test for Count Data
##
## data:  x
## p-value = 0.4615
## alternative hypothesis: true odds ratio is not equal to 1
## 95 percent confidence interval:
##  0.02991422        Inf
## sample estimates:
## odds ratio
##        Inf
```

```
gr <- split(Data3$`on treatment`, Data3$Groups)
results <- lapply(gr, Table.Frequencies)
data.frame(results)
```

```
##   M.Bovis.Niveles M.Bovis.Freq M.Bovis.Freq_Rela M.Bovis.Freq_Acum
## 1             yes            1        0.08333333                 1
## 2              no           11        0.91666667                12
##   M.Bovis.Freq_Rela_Acum M.TB.Niveles M.TB.Freq M.TB.Freq_Rela M.TB.Freq_Acum
## 1             0.08333333          yes         0              0              0
## 2             1.00000000           no        14              1             14
##   M.TB.Freq_Rela_Acum
## 1                   0
## 2                   1
```

```
table(Data3$failure, Data3$Groups)
```

```
##
##       M.Bovis M.TB
##   yes       2    2
##   no       10   12
```

```
x<-table(Data3$failure, Data3$Groups)
fisher.test(x)
```

```
##
##  Fisher's Exact Test for Count Data
##
## data:  x
## p-value = 1
## alternative hypothesis: true odds ratio is not equal to 1
## 95 percent confidence interval:
##   0.07375768 19.27169094
## sample estimates:
## odds ratio
##   1.191575
```

```
gr <- split(Data3$failure, Data3$Groups)
results <- lapply(gr, Table.Frequencies)
data.frame(results)
```

```
##   M.Bovis.Niveles M.Bovis.Freq M.Bovis.Freq_Rela M.Bovis.Freq_Acum
## 1             yes            2         0.1666667                 2
## 2              no           10         0.8333333                12
##   M.Bovis.Freq_Rela_Acum M.TB.Niveles M.TB.Freq M.TB.Freq_Rela M.TB.Freq_Acum
## 1              0.1666667          yes         2      0.1428571              2
## 2              1.0000000           no        12      0.8571429             14
##   M.TB.Freq_Rela_Acum
## 1           0.1428571
## 2           1.0000000
```

```r
table(Data3$`outcome relapse`, Data3$Groups)
```

```
##
##       M.Bovis M.TB
##   yes       0    0
##   no       12   14
```

```r
x<-table(Data3$`outcome relapse`, Data3$Groups)
fisher.test(x)
```

```
##
##  Fisher's Exact Test for Count Data
##
## data:  x
## p-value = 1
## alternative hypothesis: true odds ratio is not equal to 1
## 95 percent confidence interval:
##    0 Inf
## sample estimates:
## odds ratio
##          0
```

```r
gr <- split(Data3$`outcome relapse`, Data3$Groups)
results <- lapply(gr, Table.Frequencies)
data.frame(results)
```

```
##   M.Bovis.Niveles M.Bovis.Freq M.Bovis.Freq_Rela M.Bovis.Freq_Acum
## 1             yes            0                 0                 0
## 2              no           12                 1                12
##   M.Bovis.Freq_Rela_Acum M.TB.Niveles M.TB.Freq M.TB.Freq_Rela M.TB.Freq_Acum
## 1                      0          yes         0              0              0
## 2                      1           no        14              1             14
##   M.TB.Freq_Rela_Acum
## 1                   0
## 2                   1
```

```r
table(Data3$`lost of follow up`, Data3$Groups)
```

```
##
##       M.Bovis M.TB
##   yes       1    3
##   no       11   11
```

```r
x<-table(Data3$`lost of follow up`, Data3$Groups)
fisher.test(x)
```

```
##
##  Fisher's Exact Test for Count Data
##
## data:  x
```

```
## p-value = 0.5983
## alternative hypothesis: true odds ratio is not equal to 1
## 95 percent confidence interval:
##  0.005835461 5.120541458
## sample estimates:
## odds ratio
##  0.3468369
```

```
gr <- split(Data3$`lost of follow up`, Data3$Groups)
results <- lapply(gr, Table.Frequencies)
data.frame(results)
```

```
##   M.Bovis.Niveles M.Bovis.Freq M.Bovis.Freq_Rela M.Bovis.Freq_Acum
## 1             yes            1        0.08333333                 1
## 2              no           11        0.91666667                12
##   M.Bovis.Freq_Rela_Acum M.TB.Niveles M.TB.Freq M.TB.Freq_Rela M.TB.Freq_Acum
## 1             0.08333333          yes         3      0.2142857              3
## 2             1.00000000           no        11      0.7857143             14
##   M.TB.Freq_Rela_Acum
## 1           0.2142857
## 2           1.0000000
```

```
table(Data3$death, Data3$Groups)
```

```
##
##       M.Bovis M.TB
##   yes       1    2
##   no       11   12
```

```
x<-table(Data3$death, Data3$Groups)
fisher.test(x)
```

```
##
##  Fisher's Exact Test for Count Data
##
## data:  x
## p-value = 1
## alternative hypothesis: true odds ratio is not equal to 1
## 95 percent confidence interval:
##   0.00849267 12.15859531
## sample estimates:
## odds ratio
##  0.5579017
```

```
gr <- split(Data3$death, Data3$Groups)
results <- lapply(gr, Table.Frequencies)
data.frame(results)
```

```
##   M.Bovis.Niveles M.Bovis.Freq M.Bovis.Freq_Rela M.Bovis.Freq_Acum
## 1             yes            1        0.08333333                 1
## 2              no           11        0.91666667                12
```

```
##   M.Bovis.Freq_Rela_Acum M.TB.Niveles M.TB.Freq M.TB.Freq_Rela M.TB.Freq_Acum
## 1             0.08333333          yes         2      0.1428571              2
## 2             1.00000000           no        12      0.8571429             14
##   M.TB.Freq_Rela_Acum
## 1           0.1428571
## 2           1.0000000
```

```
normality<-function(x){
  car::qqPlot(x)
  boxplot(x)
  a<-shapiro.test(x)
  b<-FSA::ksTest(x,"pnorm", mean(x), sd(x))
  print(a)
  print(b)
  }
```

```
normality(Data3$hospitalization)
```

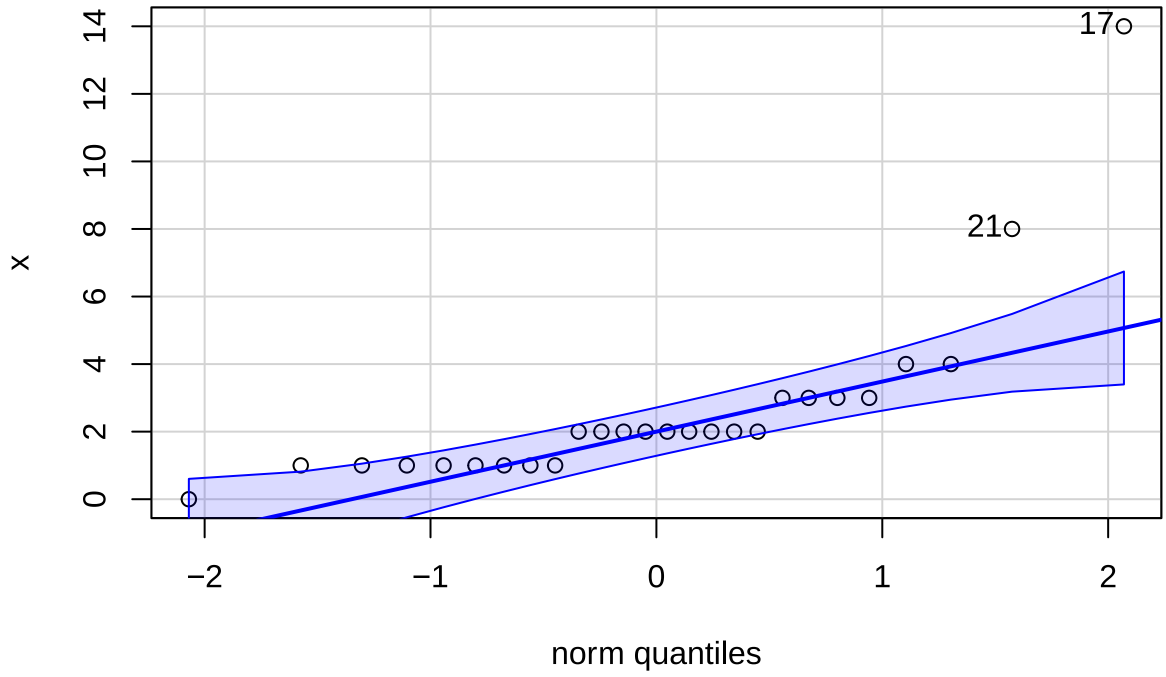


```
## Registered S3 methods overwritten by 'FSA':
##   method       from
##   confint.boot car
##   hist.boot    car

## Warning in stats::ks.test(x, y, ..., alternative = alternative, exact = exact):
## ties should not be present for the Kolmogorov-Smirnov test
```

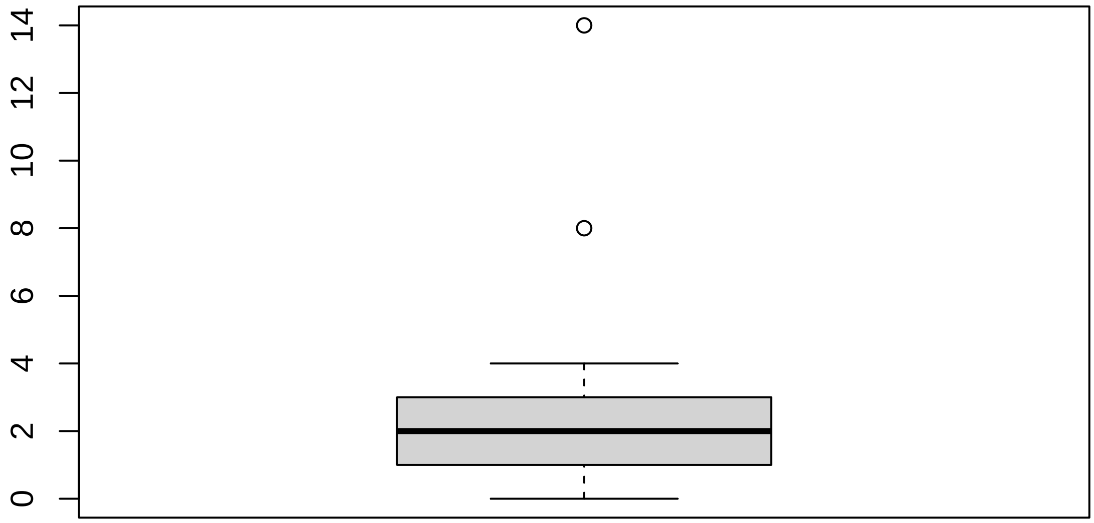


```
##
##  Shapiro-Wilk normality test
##
## data:  x
## W = 0.6154, p-value = 4.472e-07
##
##
##  One-sample Kolmogorov-Smirnov test
##
## data:  x
## D = 0.29127, p-value = 0.02427
## alternative hypothesis: two-sided
```

```
normality(Data3$`days in hospital`)
```

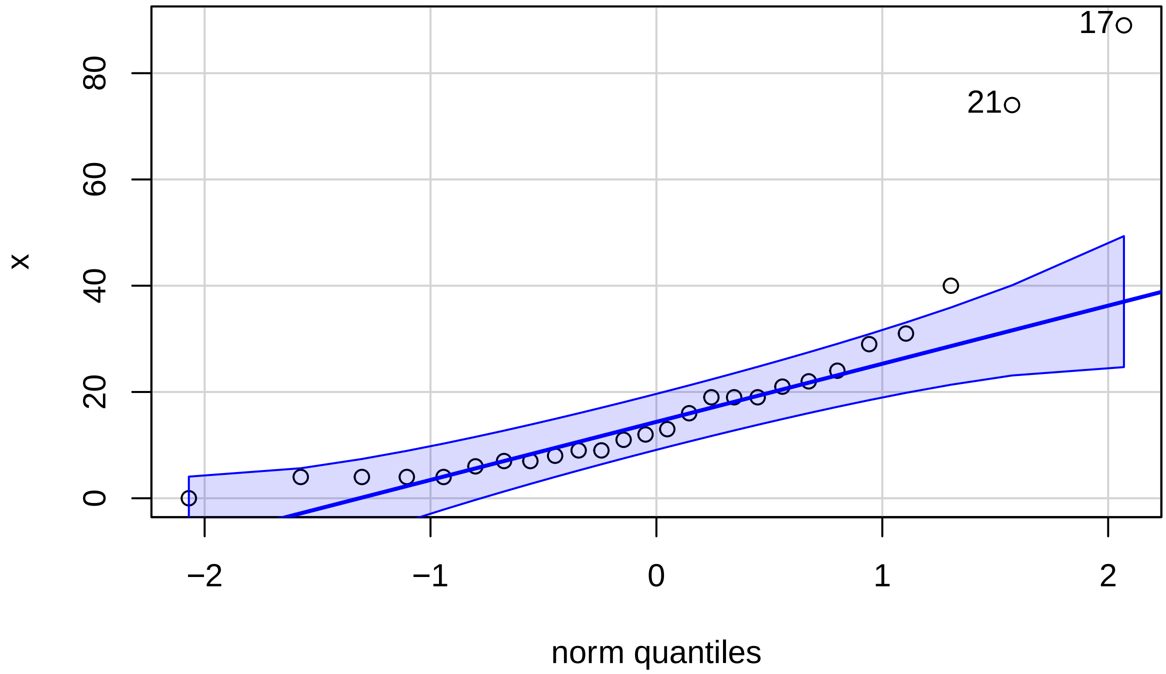


```
## Warning in stats::ks.test(x, y, ..., alternative = alternative, exact = exact):
## ties should not be present for the Kolmogorov-Smirnov test
```

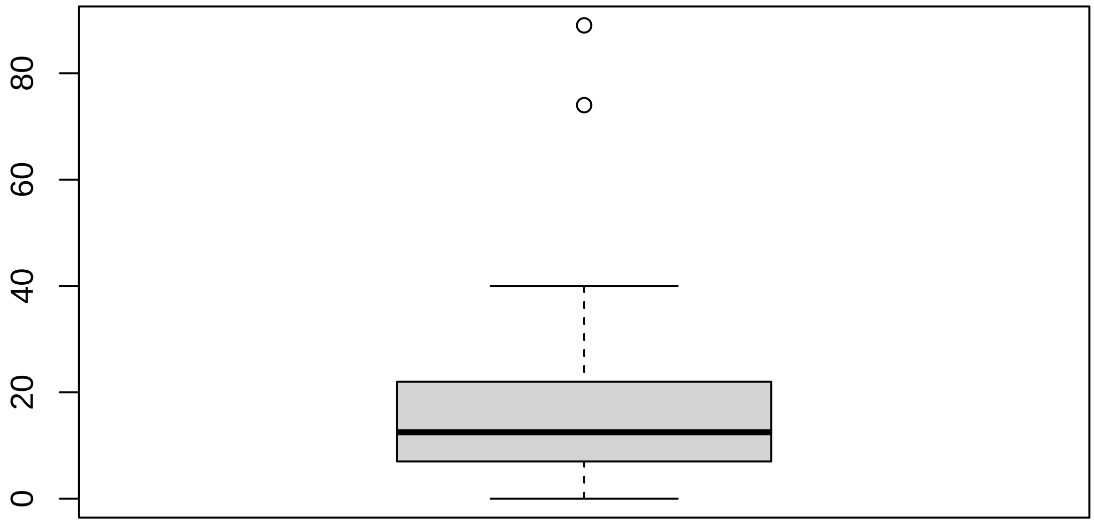

```
##
##  Shapiro-Wilk normality test
##
## data:  x
## W = 0.72417, p-value = 1.157e-05
##
##
##  One-sample Kolmogorov-Smirnov test
##
## data:  x
## D = 0.21784, p-value = 0.1695
## alternative hypothesis: two-sided
```

```
no.norm.analysis <- function(x, y){
  a <- FSA::Summarize(x ~ y)
  b <- wilcox.test(x ~ y, paired = F)
  cat("\nDescriptivos:\n")
  print(a)
  cat("\nU Mann Whitney:\n")
  print(b)
  boxplot(x~y)
}
```

```
no.norm.analysis(Data3$hospitalization, Data3$Groups)
```

```
## Warning in wilcox.test.default(x = c(0, 4, 1, 1, 3, 2, 1, 2, 1, 3, 1, 2), :
## cannot compute exact p-value with ties
```

```
##
## Descriptivos:
##          y  n     mean       sd min Q1 median   Q3 max percZero
## 1 M.Bovis 12 1.750000 1.138180   0  1    1.5 2.25   4 8.333333
## 2    M.TB 14 3.357143 3.543296   1  2    2.0 3.00  14 0.000000
##
## U Mann Whitney:
##
##  Wilcoxon rank sum test with continuity correction
##
## data:  x by y
## W = 57, p-value = 0.1568
## alternative hypothesis: true location shift is not equal to 0
```

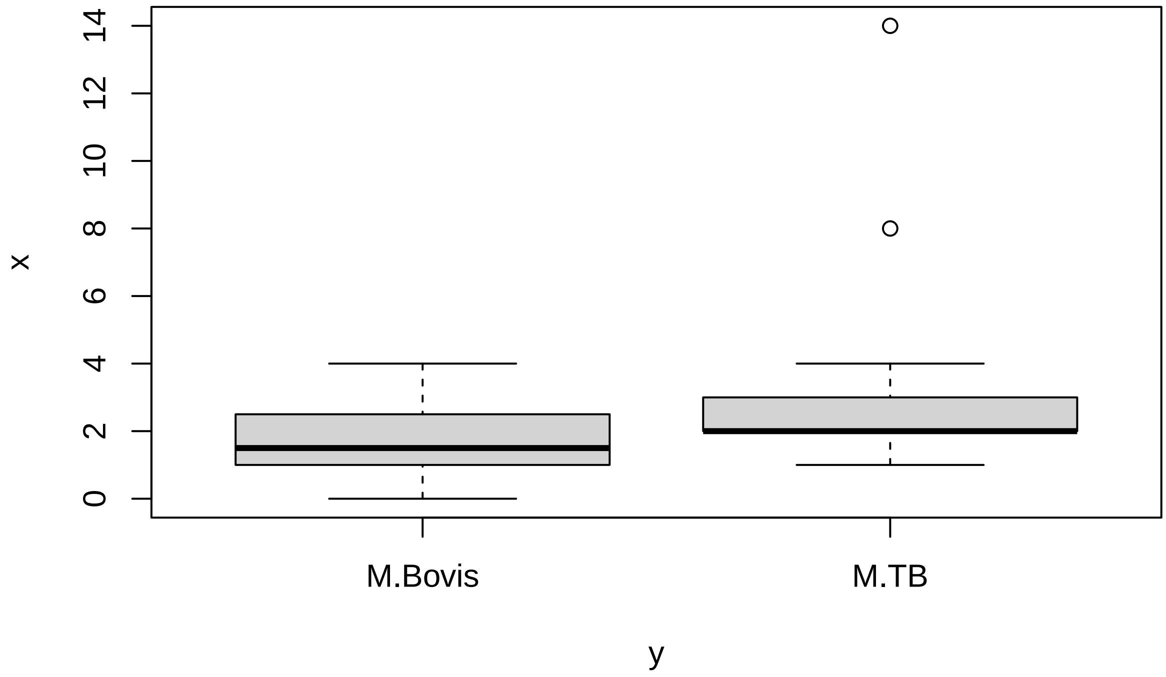


```
no.norm.analysis(Data3$`days in hospital`, Data3$Groups)
```

```
## Warning in wilcox.test.default(x = c(0, 31, 19, 29, 24, 7, 4, 11, 6, 40, :
## cannot compute exact p-value with ties
```

```
##
## Descriptivos:
```

```
##          y  n     mean       sd min   Q1 median    Q3 max percZero
## 1 M.Bovis 12 15.83333 12.59750   0 6.75   11.5 25.25  40 8.333333
## 2    M.TB 14 22.21429 26.06849   4 8.25   14.5 20.50  89 0.000000
##
## U Mann Whitney:
##
##  Wilcoxon rank sum test with continuity correction
##
## data:  x by y
## W = 79.5, p-value = 0.8366
## alternative hypothesis: true location shift is not equal to 0
```

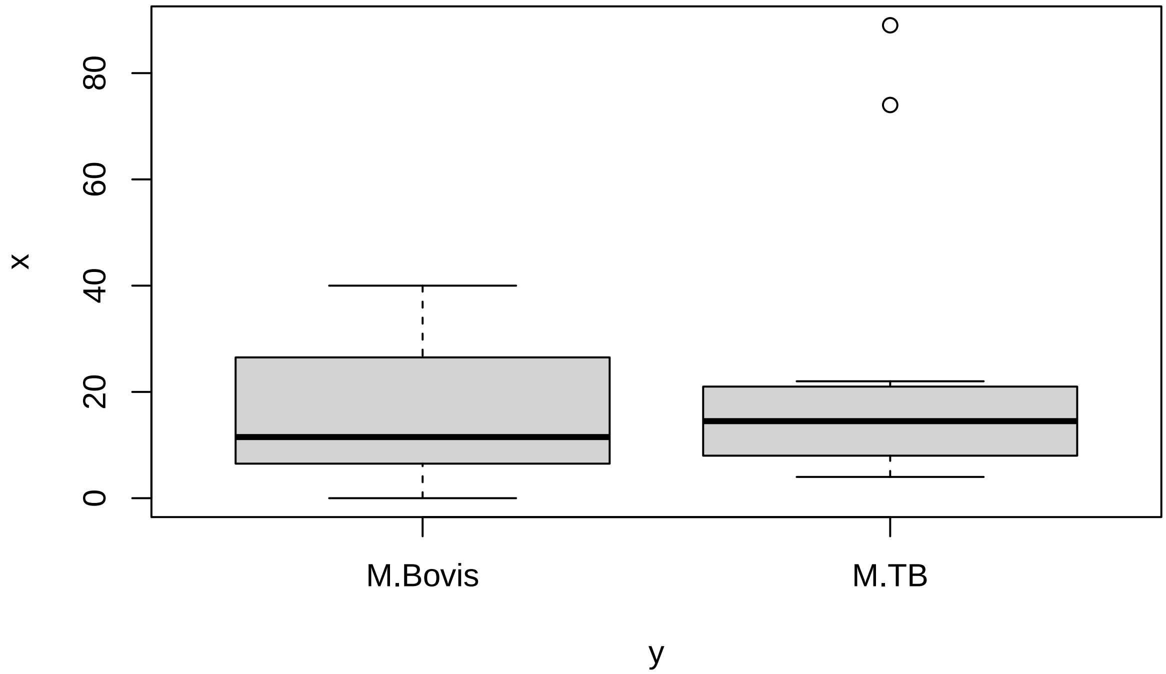

Paragraph

Crosstabs

Notes

Output Created		05-JUN-2025 12:59:01
Comments		
Input	Active Dataset	DataSet3
	Filter	<none>
	Weight	<none>
	Split File	<none>
	N of Rows in Working Data File	26
Missing Value Handling	Definition of Missing	User-defined missing values are treated as missing.
	Cases Used	Statistics for each table are based on all the cases with valid data in the specified range(s) for all variables in each table.
Syntax		CROSSTABS<br>/TABLES=grupo BY Solopulmonar soloextrapulmonar pulomarextrapulmonar<br>/FORMAT=AVALUE TABLES<br>/STATISTICS=CHISQ PHI LAMBDA UC<br>/CELLS=COUNT COLUMN<br>/COUNT ROUND CELL.
Resources	Processor Time	00:00:00.07
	Elapsed Time	00:00:00.00
	Dimensions Requested	2
	Cells Available	524245

[DataSet3]

Case Processing Summary

	Cases					
	Valid		Missing		Total	
	N	Percent	N	Percent	N	Percent
grupo * Solo pulmonar	26	100.0%	0	0.0%	26	100.0%
grupo * solo extrapulmonar	26	100.0%	0	0.0%	26	100.0%
grupo * pulomar-extrapulmonar	26	100.0%	0	0.0%	26	100.0%

grupo * Solo pulmonar

Crosstab

			Solo pulmonar		Total
			si	no	
grupo	bovis	Count	0	12	12
		% within Solo pulmonar	0.0%	60.0%	46.2%
	m tb	Count	6	8	14
		% within Solo pulmonar	100.0%	40.0%	53.8%
Total		Count	6	20	26
		% within Solo pulmonar	100.0%	100.0%	100.0%

Chi-Square Tests

	Value	df	Asymptotic Significance (2-sided)	Exact Sig. (2-sided)	Exact Sig. (1-sided)
Pearson Chi-Square	6.686[a]	1	.010		
Continuity Correction[b]	4.489	1	.034		
Likelihood Ratio	8.969	1	.003		
Fisher's Exact Test				.017	.013
Linear-by-Linear Association	6.429	1	.011		
N of Valid Cases	26				

a. 2 cells (50.0%) have expected count less than 5. The minimum expected count is 2.77.

b. Computed only for a 2x2 table

grupo * solo extrapulmonar

Crosstab

			solo extrapulmonar		
			si	no	Total
grupo	bovis	Count	5	7	12
		% within solo extrapulmonar	62.5%	38.9%	46.2%
	m tb	Count	3	11	14
		% within solo extrapulmonar	37.5%	61.1%	53.8%
Total		Count	8	18	26
		% within solo extrapulmonar	100.0%	100.0%	100.0%

Chi-Square Tests

	Value	df	Asymptotic Significance (2-sided)	Exact Sig. (2-sided)	Exact Sig. (1-sided)
Pearson Chi-Square	1.242[a]	1	.265		
Continuity Correction[b]	.474	1	.491		
Likelihood Ratio	1.248	1	.264		
Fisher's Exact Test				.401	.246
Linear-by-Linear Association	1.195	1	.274		
N of Valid Cases	26				

a. 2 cells (50.0%) have expected count less than 5. The minimum expected count is 3.69.

b. Computed only for a 2x2 table

grupo * pulomar-extrapulmonar

Crosstab

			pulomar-extrapulmonar		
			si	no	Total
grupo	bovis	Count	7	5	12
		% within pulomar-extrapulmonar	53.8%	38.5%	46.2%
	m tb	Count	6	8	14
		% within pulomar-extrapulmonar	46.2%	61.5%	53.8%
Total		Count	13	13	26
		% within pulomar-extrapulmonar	100.0%	100.0%	100.0%

Chi-Square Tests

	Value	df	Asymptotic Significance (2-sided)	Exact Sig. (2-sided)	Exact Sig. (1-sided)
Pearson Chi-Square	.619[a]	1	.431		
Continuity Correction[b]	.155	1	.694		
Likelihood Ratio	.622	1	.430		
Fisher's Exact Test				.695	.348
Linear-by-Linear Association	.595	1	.440		
N of Valid Cases	26				

a. 0 cells (.0%) have expected count less than 5. The minimum expected count is 6.00.

b. Computed only for a 2x2 table
